# Lactic Acid Bacteria – A Promising Tool for Controlling Chicken *Campylobacter* Infection

**DOI:** 10.3389/fmicb.2021.703441

**Published:** 2021-09-28

**Authors:** Agnieszka Krystyna Wyszyńska, Renata Godlewska

**Affiliations:** Department of Bacterial Genetics, Institute of Microbiology, Faculty of Biology, University of Warsaw, Warsaw, Poland

**Keywords:** microbiome, probiotic, poultry, lactic acid bacteria, *Campylobacter*

## Abstract

Since 2005, campylobacteriosis has been the most common zoonotic disease in Europe. The main reservoir of pathogenic *Campylobacter* strains is broilers, which makes raw and undercooked poultry meat two major sources of disease. Infection in chicken flocks is most often asymptomatic, despite a high level of colonization reaching 10^6^–10^9^cfu/g in animal ceca. It is widely believed that controlling the level of colonization of the birds’ digestive tract by pathogenic strains is a good way to increase food safety. Many treatments have been proposed to combat or at least reduce the level of colonization in animals reservoirs: probiotics, bacteriophages, vaccines, and anti-*Campylobacter* bacteriocins. This review focuses on the effects of *Campylobacter* infection on the chicken microbiome and colonization control strategies using probiotics (mostly lactic acid bacteria, LAB), which are live microorganisms included in the diet of animals as feed additives or supplements. Probiotics are not only an alternative to antibiotics, which were used for years as animal growth promoters, but they also constitute an effective protective barrier against excessive colonization of the digestive system by pathogenic bacteria, including *Campylobacter*. Moreover, one of the many beneficial functions of probiotics is the ability to manipulate the host’s microbiota. Recently, there have also been some promising attempts to use lactic acid bacteria as a delivery system of oral vaccine against *Campylobacter*. Recombinant LAB strains induce primarily a mucosal immune response against foreign antigens, accompanied by at most a low-level immune response against carrier strains. Since the main barrier against the invasion of pathogens in the gastrointestinal tract is the intestinal mucosal membrane, the development of effective oral vaccines to protect animals against enteric infection is very reasonable.

## Introduction

Global population growth and the improvement of the economic situation around the world are causing an increased demand for meat. It is satisfied mostly by poultry and pork, whose consumption in 2017 exceeded 120 million tons (Scanes, 2007; Mordor and Intelligence, 2018). Attaining this scale was linked to significant changes in the livestock sector in recent decades. Traditional meat production methods were gradually replaced by industrial meat production, in which animals are kept indoors for most or all of the time. This negatively affects the intestinal microbiota of animals, resulting in a reduction of resistance to pathogens, impaired absorption of nutrients, increased mortality, and nutrition costs. In the past, a common solution to these problems was a regular administration of antibiotics (Dibner and Richards, 2005). Antimicrobials given to animals effectively reduced the development of pathogens, lowering the prevalence rate of gastrointestinal infections. Antimicrobial use has also led to an increase in the level of feed use by animals, increasing growth, and meat mass. On the other hand, the extended antibiotics dosing weaken a structure of the intestinal epithelium and function of the immune system, which can result in an increased incidence of diarrhea and mortality among animals (Morgun et al., 2015). Moreover, antibiotics at sub-therapeutic doses are known to induce the development of antibiotic resistance in both commensal and pathogenic bacteria. Commensals (e.g., *E. coli*) then serve as a long-lasting reservoir of antibiotic resistance genes that can be transferred to pathogens, including those dangerous to humans (Salyers et al., 2004; Andersson and Hughes, 2014; Ma et al., 2016). It has also been reported that antibiotic growth promoters administered to chickens (oxytetracycline, erythromycin, tylosin, bacitracin, and neomycin sulfate) increased the prevalence of Proteobacteria, which include a wide variety of human pathogens, such as *Escherichia*, *Campylobacter*, *Salmonella*, and *Helicobacter* (Looft et al., 2012; Salaheen et al., 2017).

Concerns over environmental and public health risks associated with the emergence of antibiotic resistance in zoonotic bacterial pathogens due to therapeutic and/or non-therapeutic use of antibiotics have led to a global interest in adopting more stringent use of antibiotics in food animal production. In the European Union (EU), the use of antibiotics as growth promoters (AGPs) in animal feeds has been banned since 2006 (Regulation (EC) No 1831/2003 of the European Parliament and of the Council of September 22, 2003 on additives for use in animal nutrition; Maron et al., 2013). At present, antibiotics may be given to animals only in justified cases – upon recommendation and under control of a veterinarian. Despite these restrictions, overall sales of veterinary antimicrobial agents in 31 European countries in 2018 reached 6.5 thousand tons (EMA, 2020). For comparison, according to the FDA (Food and Drug Administration) by 2019, 11.46thousand tons of antibiotics were sold in the United States for livestock (FDA, 2019). ESVAC (The European Surveillance of Veterinary Antimicrobial Consumption) and FDA reports show that 15 and 13.15% of these antibiotics were used in the European and US poultry industries, respectively. This was one of the reasons for an increasing pressure for stricter regulations in North America. The regulation issued by the US FDA that came into force on January 1, 2017 banned the use of antibiotics for enhancing growth in livestock (FDA, 2015). This rule prohibits the over-the-counter sale to farmers of antimicrobial drugs medically important for humans (EMA, 2017).

Reduced growth rates in animals that are observed in the absence of AGPs will impact the efficiency of production and perhaps jeopardize food security. It has also been reported that the ban on AGPs in poultry feed may lead to an increase in the therapeutic use of antibiotics, with enteric diseases and necrotic enteritis in particular as major indications (Hughes et al., 2008). Therefore, it became necessary to develop novel alternatives to growth stimulants that could strengthen the natural defenses of animals and thus prevent the expansion of pathogenic microflora in the gastrointestinal tract and at the same time exert a positive effect on animal breeding traits. That is the reason why the interest in probiotics and their possible use as food additives for animals has increased significantly in recent years.

## Campylobacteriosis

Among the foodborne diseases, zoonoses, which are infections transmitted from animals to humans (either directly or *via* the food chain), are of great importance. Studies indicate that between a third and up to a half of all human infectious diseases have a zoonotic origin (EFSA, 2016). Since 2005, campylobacteriosis has been the most commonly diagnosed zoonosis in the residents of the EU Member States. According to data from the European Food Safety Authority in EFSA (2016), 220,682 cases of *Campylobacter* infections were confirmed, with an incidence rate 59.7 per 100,000 (EFSA and ECDC, 2021). However, since most infections are mild, only one in 47 cases of campylobacteriosis is reported; thus, the number of *Campylobacter* infections is grossly underestimated (Havelaar et al., 2013).

Campylobacteriosis is a result of infection with thermotolerant *Campylobacter* bacterial strains. Although new species of *Campylobacter* have been recently discovered, human cases of campylobacteriosis are dominated by two main species, *Campylobacter jejuni* and, to a lesser extent, *Campylobacter coli*. Limited data are available on infections caused by other species, i.e., *Campylobacter lari* or *Campylobacter fetus* (Kaakoush et al., 2015). The course of *Campylobacter* infection depends on the pathogenicity of the strain and on the responsivity of the host’s immune system. The clinical manifestation is variable and ranges from asymptomatic to acute intestinal inflammation accompanied by a long-lasting, mucoid diarrhea. Symptoms usually resolve spontaneously after about 7days; however, *Campylobacter* infections sometimes lead to the development of autoimmune and neurological diseases, examples of which are reactive arthritis and neuropathy of the peripheral nervous system, i.e., Guillain-Barré syndrome (Dasti et al., 2010; Goodfellow and Willison, 2016). Current studies indicate that *Campylobacter* infections may also be associated with irritable bowel syndrome (IBS) and colorectal cancer (Kaakoush et al., 2015). A small percentage of patients with *Campylobacter*-induced enterocolitis develop bacteremia (Robyn et al., 2015).

Although the infection may be also caused by the consumption of water, unpasteurized milk or beef contaminated by pathogens, epidemiological studies show that the majority of cases of campylobacteriosis is caused by the consumption of infected, inadequately prepared poultry, consistent with the observation that the main reservoir of *Campylobacter* is farm poultry and wild birds (Dasti et al., 2010; Silva et al., 2011). According to the report in EFSA (2016), as many as 29.6% of the tested fresh broiler carcasses in Europe were contaminated with *Campylobacter* (EFSA and ECDC, 2021). This figure is lower than in previous years, when on average 38% of the meat samples contained *Campylobacter*. Notably, the rate of *Campylobacter*-positive samples of broiler meat varied greatly between individual members of the European Union (EFSA and ECDC, 2017, 2021). It is important to note that the data from various investigations often are not directly comparable because of differences in sampling strategies and testing methods, including sampling season (in most countries, *Campylobacter* infections are known to be more prevalent in poultry during the summer than during the winter).

A large number of cases, the occurrence of post-infectious complications (mainly neurological ones), as well as the growing prevalence of *Campylobacter* strains resistant to antibiotics make campylobacteriosis a serious problem for medical services. *Campylobacter*, mainly due to its antibiotic resistance, forced its way to the list of bacterial species constituting the greatest threat to human health, published 2017 by the World Health Organization (WHO).^1^ Therefore, in recent years, researchers focused on developing a strategy to prevent *Campylobacter* infections. It seems that controlling the level of colonization of the digestive tract of animals should improve food safety. For example, it has been determined that a reduction in the *C. jejuni* chicken carcass contamination by 2 log would reduce the risk of campylobacteriosis in humans 30-fold (Rosenquist et al., 2003; EFSA Panel on Biological Hazards (BIOHAZ), 2011). Table 1 provides various approaches that are being developed to control the *Campylobacter* infection on farms. Some of them are promising and results of using others are modest and variable.

**Table 1:** Proposed strategies to control the *Campylobacter* infection for use on farms.

Strategy	Preventive measures against *Campylobacter* infection	Measures to reduce *Campylobacter* infection
Good hygienic practices and biosecurity	Hald et al., 2007	Lin, 2009
Treatment of drinking water	Chaveerach et al., 2002	Byrd et al., 2001
Diet modification (antimicrobial additives in water and food)	Guyard-Nicodeme et al., 2016	Hilmarsson et al., 2006; De Los Santos et al., 2009
Vaccination	Buckley et al., 2010; Layton et al., 2011; Kobierecka et al., 2016b;Nothaft et al., 2016	–
Passive immunization	Sahin et al., 2003; Vandeputte et al., 2019	Tsubokura et al., 1997; Hermans et al., 2014
Bacteriophage therapy	–	Loc Carrillo et al., 2005; Wagenaar et al., 2005; Kittler et al., 2013
Bacteriocin	–	Stern et al., 2005, 2006; Zommiti et al., 2016
Probiotic	Arsi et al., 2015; Manes-Lazaro et al., 2017	Morishita et al., 1997; Neal-McKinney et al., 2012
Prebiotic	Baurhoo et al., 2009; Kim et al., 2019	–
Genetic selection of chicken (breeding of chickens lines resistant to *Campylobacter* colonization)	Boyd et al., 2005; Kaiser et al., 2009; Swaggerty et al., 2017	–

## Microbiome of Chickens

Studies of various animal species, including humans, have shown that the intestinal microbiota has a huge impact on the health of the host, and the disturbance of its balance (dysbiosis) is associated with the occurrence of various diseases, such as inflammatory bowel disease, IBS, obesity, and diabetes (Turnbaugh et al., 2006). Since the ban on AGP in animal feed, these dysbiosis-related problems have become a major issue, especially in intensive animal farming.

The gut microbiome of a healthy animal is quite stable, although its composition and activity depend on many factors. It may change as a result of viral or bacterial infections or of treatment with antimicrobials (Ley et al., 2008; Yegani and Korver, 2008; Costa et al., 2015; Kumar et al., 2018). It is also determined by zoohygienic conditions, age (Mueller et al., 2006; Williams et al., 2013), health status (Abt and Artis, 2009), mental stress, and genetic factors (Meng et al., 2014; Mandal et al., 2020).

The digestive tract of poultry hosts at least 900 species of microorganisms (Apajalahti et al., 2004). The majority of them belong to a commensal microflora, which stimulates the production of cytokines (i.e., tumor necrosis factor) and through them, impacts on the activity of lymphocytes and other mucosal subpopulations of host immune cells (Atarashi and Honda, 2011; Meijerink et al., 2020). The intestinal bacteria are also of high importance for the gut barrier function as they regulate the proliferation and differentiation of intestinal epithelial cells (Pan and Yu, 2014). The metabolic abilities of bacteria forming the intestinal microbiome enable the degradation of complex food substrates (e.g., plant cell wall components). The final products of the fermentation of the resulting simple sugars are short-chain fatty acids (SCFA), which become an important source of energy and carbon for the host (Sergeant et al., 2014).

The number of pathogenic microorganisms in the digestive tract of a healthy farm animal is usually low, constantly controlled by other microorganisms in the intestinal ecosystem and does not pose a serious threat to the host health. Intestinal microbiota contributes to the host defense in multiple ways, but one of the most important ones is called a “competitive exclusion.” In healthy animals, commensal bacterial communities in the GI tract colonize intestinal mucosa and form a layer covering the mucosal surface. This layer of microbial communities can effectively block the attachment and subsequent colonization by most invading enteric pathogens (Lan et al., 2005). Moreover, changes of the intestinal pH, modulation of the oxygen level, and the utilization of nutrients carried out by intestinal microbiota help to generate an unfavorable environment to pathogens (Sekirov et al., 2010). Thus, a stable healthy gut microbiota is an effective barrier against the colonization of pathogens. The microbial community has also an important role in modulating the host immune system, maintaining normal physiological homeostasis, and influencing host metabolism (Sommer and Backhed, 2013). Thus, all alterations in its composition may have adverse effects on birds’ health and on an efficiency of energy extraction from feed.

The development of high-throughput sequencing approaches provided an opportunity for an in-depth investigation on the taxonomic composition of the poultry intestinal microbiome. It seems that understanding how the gut microbiota of chickens is shaped will help in the development of effective probiotics or other successful interventions aimed at chicken’s health.

In the majority of poultry farms, microorganisms that form the microbiome of the chicks’ digestive system come from the breeding environment. That is, why large differences in microbiota composition are observed immediately after hatching (Pedroso et al., 2005). Since farm chickens do not have contact with adult birds, re-use of litter is a common practice in the production of broilers. Cressman et al. demonstrated that the ileal mucosal microbiome of chickens reared on fresh litter was dominated by *Lactobacillus* spp., whereas a group of unclassified *Clostridiales* was the dominating bacteria in chickens reared on reused litter (Cressman et al., 2010).

**Figure 1:**
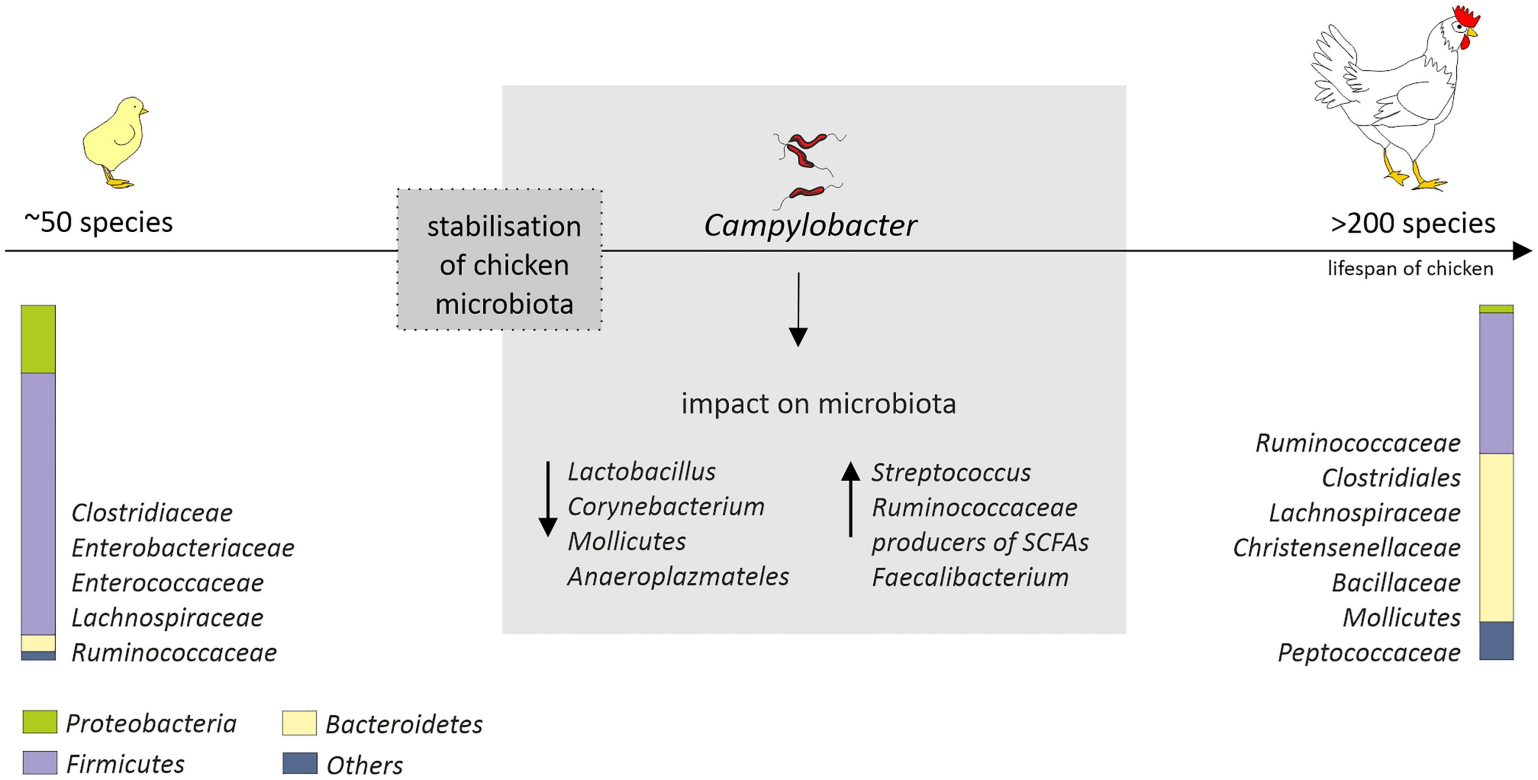
Age-related changes in the chicken cecal microbiota (from hatch to 42days of age) and potential impact of *Campylobacter jejuni* colonization that could affect microbiota composition. After hatch, the microbiota is mainly composed of environmental bacteria. Stabilization of the microbial diversity occurs after 10–20days post-hatch. The diagram is based on the information provided by (Oakley et al., 2014a; Thibodeau et al., 2015; Connerton et al., 2018; Richards et al., 2019; Duquenoy et al., 2020). The distribution of the most common and abundant bacterial taxa (phylum, order, and family) in the ceca of the chickens is presented.

The “natural” core microbiome of broiler chickens is difficult to define, and this is the consequence of a high variability not only between birds, but also between whole flocks or even breeds (Pandit et al., 2018; Richards et al., 2019). Nevertheless, the short transit time of food, a consequence of the shortness of the gastrointestinal system of birds, promotes bacteria that adhere to the mucosal layer and/or grow fast (Pan and Yu, 2014). The most diverse microbiome is the cecal one. This is favored by the longest feed retention time (12–20h; Singh et al., 2012). The cecum is dominated by representatives of the *Clostridiaceae*, *Bacteroidaceae*, *Lactobacillaceae*, and *Lachnospiraceae* families (Witzig et al., 2015; Richards et al., 2019). The diversity and distribution of bacterial species that make up the GIT microbiota initially fluctuate but become well established as soon as by day 3 post-hatch (Apajalahti et al., 2004) or, in another study, by day 11 (van der Wielen et al., 2002). In turn, a rapid increase in diversity up to day 12 with variation observed both in terms of genera and abundance, before the stabilization of the microbial diversity after day 20 was observed by Ijaz et al. (2018). On the 42nd day of life of chickens over 200 species were identified in their intestines, while after hatching, only 50 were found (Oakley et al., 2014a). Initially, the intestinal microbiome is dominated by Gram-negative bacteria, in particular *Enterobacteriaceae* (*Salmonella*, *Klebsiella*, *Proteus*, and *E. coli*). In week-old chicks, representatives of *Firmicutes* (*Lachnospiraceae, Ruminococcaceae, Clostridiales*, *Christensenellaceae, and Bacillaceae*) and *Bacteroidetes* (*Bacteroidaceae*) emerge (Ballou et al., 2016; Kumar et al., 2018; Richards et al., 2019) and gradually begin to prevail. On the 28th day of birds’ life according to the Ballou et al. study, Gram-negative bacteria account for less than 6% of the microbiome. The arrival of the SCFAs producers, i.e., *Lachnospiraceae*, *Ruminococcaceae*, and *Bacteroidaceae*, may explain the gradual decrease in the presence of *Enterobacteriaceae* in young birds’ intestines (Figure 1); thus, early interventions promoting this effect might be highly desirable.

A comparison of the microorganisms presents in the lumen of the intestine with those associated with the mucous membrane showed a much greater diversity of the latter, especially in the ileum and the cecum (Borda-Molina et al., 2016). *Pseudomonas* spp. – species that have the ability to hydrolyze phytate or to degrade starch and improve the availability of plant phosphorus – have been identified only in the mucosa of the ileum (Maougal et al., 2014). In the mucous membrane, species belonging to the genera *Clostridium* XI and *Ralstonia* were also present in large numbers, whereas *Lactobacillus* sp. strains were three times more abundant in the lumen of the ileum (Borda-Molina et al., 2018). The difference in lumen and mucous microbiota composition was also observed by Richards et al. (2019).

A close relationship between the composition of the microflora and the productivity of the poultry has been identified (Stanley et al., 2013, 2016; Clavijo and Florez, 2018; Johnson et al., 2018). Animals with a high feed conversion ratio (FCR) exhibited a higher abundance of the genera of *Acinetobacter*, *Bacteroides*, *Streptococcus*, *Faecalibacterium prausnitzii, Clostridium* (from families *Lachnospiraceae, Ruminococcaceae*, and *Erysipelotrichaceae*), and *Lactobacillus*. Simultaneously, a negative correlation between performance parameters and *Enterobacteriaceae* expansion has been reported (Singh et al., 2014; Stanley et al., 2016). Other bacterial taxa, strongly correlated with broiler chicken performance, were identified by Johnson et al. and included as: *Butyricimonas*, Candidatus division *Arthromitus*, *Faecalibacterium*, *Parabacteroides*, and *Sutterella* (Johnson et al., 2018).

Consequently, in the gut of normal, healthy, and non-stressed animals, there is a dynamic balance between beneficial and non-beneficial bacteria. On farms, this balance can be disturbed, for example, by various stress conditions which animals may be exposed to. In the poultry production systems, these include transportation, change or withdrawal of food and water, and a high density of individuals in a relatively small space. All kinds of stress, both physiological and psychological, weaken the immune system, which leads to intestinal dysfunction, increases the permeability of the intestinal barrier, and predisposes to the colonization of the digestive tract by pathogenic microorganisms (Mandal et al., 2020).

## The Effect of *Campylobacter* Infection on Chicken Microbiome

In industrialized countries, *Campylobacter* is the most common cause of bacterial foodborne infections (Hermans et al., 2012b). Two species responsible for the majority of human diseases, *C. jejuni* and *coli*, are extremely widespread in the production of poultry. *Campylobacter* mainly colonizes lower parts of the digestive tract of birds, and the level of colonization is very high, up to 10^9^cfu / gram of cecal contents (Sahin et al., 2015). Even such a high level of colonization does not cause disease symptoms in birds, which prevents the elimination of infected individuals from the flock. It has been suggested that persistent, high-level cecal colonization of *C. jejuni* in its avian host stems from an inefficiency of the chicken immune system combined with mechanisms redirecting the response toward tolerance (Hermans et al., 2012a). Most previous studies indicate that *Campylobacter* is a poultry commensal (Lee and Newell, 2006), but there are also results which report detrimental health effects associated with the colonization of chicken gastrointestinal tract by *C. jejuni* (Williams et al., 2013; Humphrey et al., 2014; Awad et al., 2015). Noticed in Humphrey et al. (2014) that broiler gut mucosa was damaged by *C. jejuni* M1 strain: high level of inflammation occurred, leading to a diarrhea and consequent poor bird welfare (Humphrey et al., 2014). The ability to induce intestinal damage and to modulate the barrier function of the intestinal epithelia facilitates the paracellular passage of *C. jejuni* into the underlying tissues and supports the translocation of luminal bacteria, such as *E. coli* to internal organs (Lamb-Rosteski et al., 2008; Kalischuk et al., 2010; Awad et al., 2015, 2016). Similar clinical symptoms have been seen by other research groups (Ghareeb et al., 2012; Awad et al., 2015, 2018). It appears that the outcome of infection depends on the genetic interplay between the host and *Campylobacter* strain.

*C. jejuni* is rarely detected in gastrointestinal tract of commercial flocks under 2weeks of age, regardless of the production system (Conlan et al., 2007; Newell et al., 2011). This suggests that a mechanism counteracting colonization of young birds by *Campylobacter* exists. It has been speculated that a high level of specific maternal antibodies provides protection from *Campylobacter* colonization (Cawthraw and Newell, 2010). Observations that chickens residing in flocks together with adults are free of *Campylobacter* for the first few weeks after hatching seem to confirm this notion (Sahin et al., 2003).

There are also reports suggesting that a strong shift in the bacterial microbiome is a leading cause for an age-dependent infection of chickens with *Campylobacter* (Han et al., 2016). But at the same time, it has been shown that chickens between 0 and 3days of age, whose microbiome is composed of species very different from those found in 2–3week old chickens, can become infected with *Campylobacter* and shed the bacterium. These observations are purely experimental and differ from those obtained in the field (Conlan et al., 2007). In turn, Conlan et al. suggest that the mechanism behind the “lag-phase” reported in commercial flocks results from the age-dependent transmission between hosts rather than from their susceptibility to colonization (Conlan et al., 2011). This is supported by studies conducted by Connerton et al., which indicate that chickens can become infected at any time during the rearing period, but the colonizing *Campylobacter* only multiplies to the extent of being detectable and efficiently transmittable when birds are over 2weeks old (Connerton et al., 2018). Recently Ijaz et al. have performed comprehensive day-to-day investigation of the chicken cecal microbiome from day 3 to 35. They identified *Campylobacter* at day 16, just after the most substantial changes in metabolic profiles observed, and hypothesized that a shift from competitive to environmental drivers of microbial community from days 12 to 20 creates a window of opportunity whereby *Campylobacter* appears (Ijaz et al., 2018).

Although the chicken microbiome is being increasingly characterized, information on the effect of foodborne pathogens on its modulation/composition is still lacking. Only a few studies have reported changes in the chicken microbiota in response to *Campylobacter* colonization.

In studies conducted by Kaakoush et al., a presence of *C. jejuni* in the chicken gastrointestinal tract was associated with a lower abundance of *Lactobacillus* and *Corynebacterium* and a higher abundance of both *Streptococcus* and *Ruminococcaceae* in fecal samples (Kaakoush et al., 2014; Figure 1). Also a presence of major producers of SCFA (*Bacteroides*, *Alistipes* and *Blautia*) was noted. Since *C. jejuni* can use organic acids produced by these genera as an energy and carbon sources, this could, at least partially, explain their co-occurrence in infected birds. In this experiment, a modulation of the microbiome was assessed in birds originating from different farms and production types. Chicken microbiome modifications induced solely by *C. jejuni* during a controlled experimental challenge was measured in the studies carried out by Thibodeau et al. (2015). They noticed a link between *C. jejuni* and *Clostridium*, which is also a producer of SCFA. They hypothesized that *C. jejuni* could act as a hydrogen sink that would improve the growth of some *Clostridium* and their competitive standing through increased fermentation, leading in consequence to increased production of organic acid that can be used by the *Campylobacter*. Variable shifts in the abundance of members of the *Clostridiales* in response to *Campylobacter* colonization were observed also by Connerton et al. (2018). However, they noticed that several clostridial OTUs (most notably *Clostridium* XIVa) show a greater an abundance in the absence of *C. jejuni*. In the human gut, representatives of this group are major butyrate-producing bacteria and play a key role in maintaining metabolic and immune functions (Lopetuso et al., 2013). The presence of *C. jejuni* in the chicken gastrointestinal tract was also associated with a diminution of the relative abundance of *Mollicutes* and *Anaeroplasmateles* (Thibodeau et al., 2015). In recently conducted studies, a high level of *Campylobacter* has been also linked to a higher abundance of *Faecalibacterium* (Duquenoy et al., 2020). The positive interaction of these species has not yet been fully explained. Butyrate produced by *Faecalibacterium* is harmful to *Campylobacter* but may also trigger the expression of *C. jejuni* genes that are important for host colonization (Duncan et al., 2004; Van Deun et al., 2008). *Faecalibacterium prausnitzii* has also been found to be involved in the modulation of mucin production by goblet cells (Wrzosek et al., 2013). The production of mucus could have a beneficial effect for *Campylobacter* as the mucous layers provide a protective niche for them, allowing to resist intestinal peristalsis and the action of organic acids. The importance of *Faecalibacterium* has also been highlighted in a recent study conducted by Patuzzi et al. Their network analysis showed that *Limnobacter*, *Parabacteroides*, *Pseudomonadaceae*, *Sutterella*, *Sphingobium*, and *Oxalobacteraceae* were positively affected by *Faecalibacterium* and *Lactobacillus*, and at the same time, a negative interaction from *Campylobacter* was detected toward them. The author hypothesized that these six taxa might be involved in the maintenance of the resilience within the microbial community (Patuzzi et al., 2021). This is in line with the theory put forward by Duquenoy et al. Increased abundance of *Faecalibacterium prausnitzii*, which has a documented anti-inflammatory effect, would improve the ability of *Campylobacter*-colonized chickens to control inflammation caused by this microorganism (Duquenoy et al., 2020). Therefore, the importance of *Faecalibacterium* for chicken intestinal health remains to be determined.

It is conceivable that the chicken cecal microbiome is not extensively disturbed by colonization with *C. jejuni*. Elucidation of the role of gut microbiota in *C. jejuni* infection in chickens thus requires a more detailed understanding of their ecology. For the time being, the role of the chicken microbiota composition for the susceptibility to *Campylobacter* infection remains elusive.

In addition to microbiota, Connerton et al. have compared inflammatory responses, and zootechnical parameters of broiler chickens not exposed to *Campylobacter* with those exposed either at young age (6days old) or at 20days old, when commercial broiler chicken flocks usually become colonized. A transient growth rate reduction was observed only during early colonization. Both early and late colonization produce pro-inflammatory responses, but their kinetics are quite different. For birds infected on day 6, first a relative increase in IFN-γ and IL-4 was observed and then expression of IL-6, IL-17A, and IL-17F increased. These pro-inflammatory cytokines declined after upregulated expression of IL-10. Ultimately, cytokines in the early colonized birds returned to levels not distinguishable from age-matched noninfected birds (Connerton et al., 2018). Cytokine expression in response to *Campylobacter* infection in chickens challenged at day 20 demonstrated the upregulation of IL-6, IL-17A, and IL-17F; elevated IL-17A response was observed until the 35day of life. These differences do not result in lower *Campylobacter* colonization levels at the end of the study but can lead to shifts in the resident microbial communities (Connerton et al., 2018).

Changes in the levels of pro-inflammatory, anti-inflammatory, and regulatory cytokines in *Campylobacter*-infected chickens were also observed by Mortada et al. A study of the effects of this pathogen on CD4+ and CD8+ cells throughout the life of chickens showed that *Campylobacter* is capable of inducing both Th1 and Th2 immune responses. The ability to maintain the balance between them might explain a high level of cecum colonization in *Campylobacter*-infected birds with no pathological changes (Mortada et al., 2021).

Developing effective probiotic-based strategies for controlling *Campylobacter* infection in chickens certainly require understanding of the interaction of this pathogen with the chicken microbiota as well as its influence on the functioning of birds’ immune system.

## Lactic Acid Bacteria as Probiotics

Probiotics are currently defined as mono or mixed cultures of live microorganisms, which – when administered in adequate amounts – confer a health benefit to the host (FAO-WHO, 2006). Their action, consisting mainly of antagonistic activity against pathogens and modulation of the immune system, is important in maintaining the balance of intestinal microflora. Most commonly used probiotic preparations contain lactic acid bacteria (LAB), although they can also be formulated using bacteria from outside of this group, as well as fungi showing beneficial effects on health (Sarao and Arora, 2017). LAB is an artificially extracted group with a huge genetic and phylogenetic diversity. These are Gram-positive, nonsporulating, catalase-negative, acid-tolerant, anaerobic or aerotolerant, and auxotrophic bacteria characterized by the ability to convert carbohydrates into lactic acid *via* fermentation. Its most numerous representatives are bacteria of the genera *Lactobacillus* and *Bifidobacterium*. This group also includes microorganisms belonging to such genera as: *Enterococcus*, *Lactococcus*, *Leuconostoc*, *Pediococcus*, and *Streptococcus*.

The LAB has been used for millennia in the production of fermented foods. They are “generally regarded as safe” (GRAS status) according to The American Food and Drug Administration. Several species, including *Lactobacillus plantarum* and *Lactobacillus fermentum*, have received a qualified presumption of safety status given by European Food Safety Authority (EFSA).

The mechanism of the beneficial effect of probiotics is not fully understood. It is primarily based on interaction with a complex microbiome on the surface of the intestinal epithelium. Probiotics compete with pathogenic microorganisms for sites of adhesion to intestinal epithelial cells. They can also produce various compounds that inhibit the growth of pathogens, such as organic acids, hydrogen peroxide and bacteriocins (small proteins with specific bactericidal activity), and compete for available nutrients, which makes the environment less permissive for the growth of pathogens (Ng et al., 2009; Bermudez-Brito et al., 2012; Peng et al., 2016; Oh et al., 2017). Probiotic bacteria also play a role in improving barrier function by enhancing the expression of genes involved in epithelial tight junction formation and by increasing synthesis of mucin that forms mucus, a layer protecting from pathogens, enzymes, toxins, dehydration, and abrasion. The positive effects of probiotics manifest themselves also through increased activity of digestive enzymes and improved breakdown of indigestible nutrients. Studies conducted so far indicate that probiotics can modulate and regulate intestinal immune responses by reducing pro-inflammatory cytokines, increasing secretory IgA production, and promoting specific and non-specific immune responses against pathogens (activation of macrophages; Haghighi et al., 2008; Ng et al., 2009; Bermudez-Brito et al., 2012; Martinez et al., 2016).

**Figure 2:**
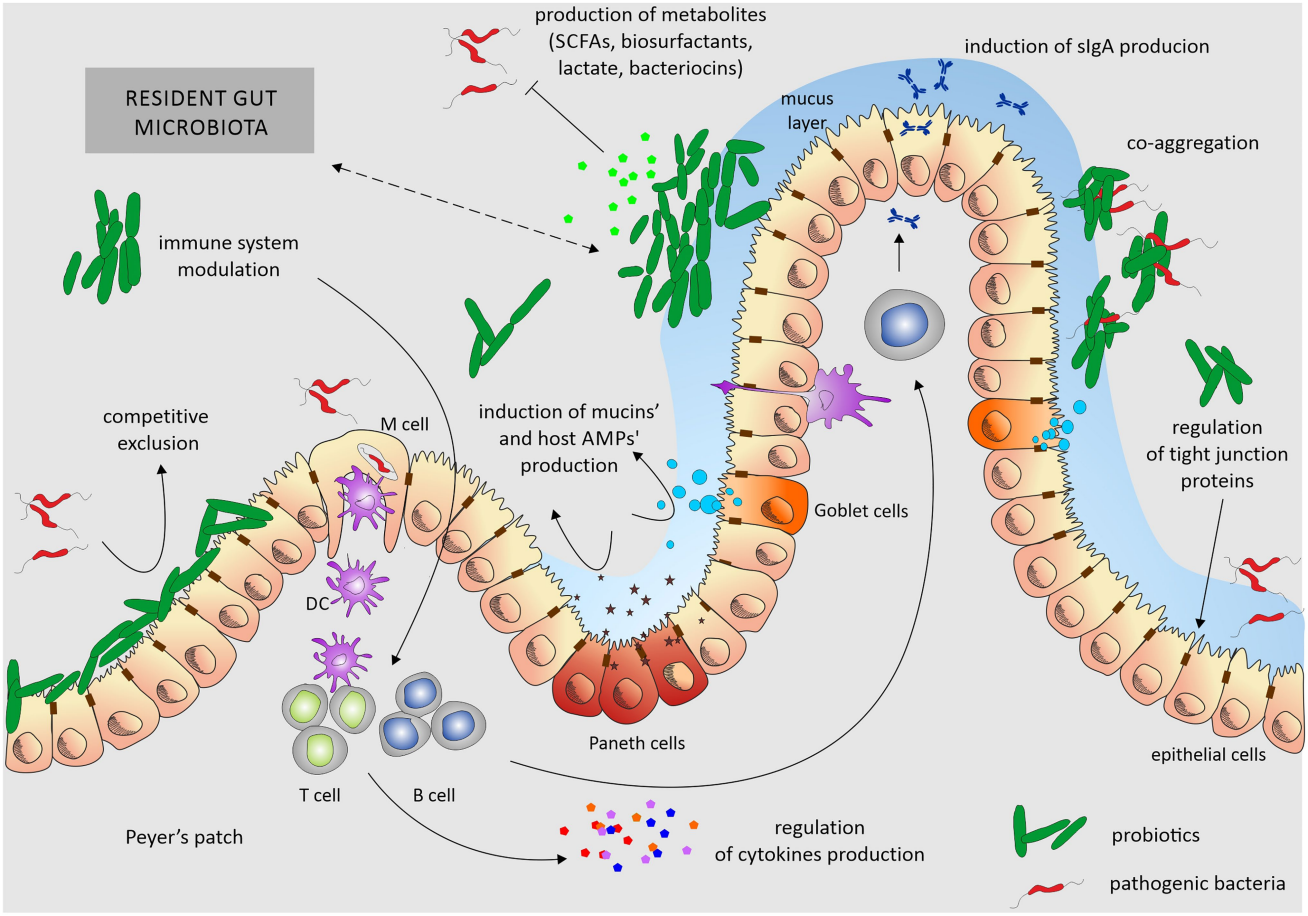
Overview of mechanisms of probiotics’ action. Probiotics exert their beneficial effects mainly by ensuring the proper balance of the microbiota colonizing the gut. Probiotic activity depends on stabilization of the epithelial barrier, induction of mucin secretion, and aggregation skills. By adhering to enterocytes, they reduce the opportunity for the colonization of this ecological niche by pathogenic bacteria. They also produce various, not always fully characterized, metabolites that inhibit the growth of pathogens. Additionally, they modulate the host immune system. Probiotics influence the gut through one or a combination of these mechanisms. Dotted arrow underlines the interplay between the host microbiota and probiotic strains and nature of these interactions can be both – positive, negative, depending on the composition of resident gut microbiota and used probiotic. DC, dendritic cells.

Several interesting reviews were published recently that describe mechanisms of probiotics action in details (Khan et al., 2020; Tarradas et al., 2020). We summarize these findings in Figure 2.

### The Impact of Probiotics on Chickens’ Microbiota

For decades, various approaches have been used in animal husbandry with aim to influence the composition of animal gut microbiome. However, due to dramatic deficiencies in former research methods, there was little understanding of the complexity of the intestinal microbial population and its relationship to animal health. The introduction of high-throughput DNA-based molecular biology techniques, such as metagenomics and new generation DNA sequencing, completely changed this picture. These methods allow for characterization of uncultivable members of intestinal microbiota, thus shedding light on the composition and temporal spatial location of the microbial population in animal intestine. One of the effects that is attributed to probiotics is to improve the health and performance of farm animals by manipulating the host microflora. In the case of broilers, it was shown that supplementation of food with probiotics is capable to accelerate the growth rate, which may be associated with increased food absorption. Another reason for a more efficient use of feed may be the production of numerous carbohydrate-degrading enzymes, such as β-glucans (He et al., 2019; Sureshkumar et al., 2021).

Manipulation of the microbiome through probiotics has long been used by the poultry industry to increase growth rates and feed conversion and to improve gut health of chickens. However, there are quite conflicting data on the actual ability of probiotics to stimulate the growth of chickens. Some studies demonstrated that supplementing feed with probiotics cultures can enhance body weight gain and feed efficiency and reduce mortality rate in broilers (Timmerman et al., 2006; Salim et al., 2013; De Cesare et al., 2017). Others observed that colonization of the lower portion of the small intestine by *Lactobacillus* strains may negatively affect the performance of chickens (Oakley et al., 2014b). And some indicate no significant effects on host feed consumption and the feed-to-growth conversion ratio (Nakphaichit et al., 2011). The positive health effect is also brought by the administration of probiotics to alleviate the side effects of antibiotic treatment. But one should keep in mind that interactions between host and microbiota are complex and may also have negative effects (Rinttila and Apajalahti, 2013).

Unfortunately, for a long time, technical limitations have made it difficult to check whether the use of live bacterial preparations affects the composition and diversity/development of the microbiome. We can now determine how these treatments affect the microbiota and the host, and this understanding will allow us to use more targeted approaches in the future. Ballou et al. (2016) characterized the microbiome of chickens that were administered live bacterial preparations commonly used in the production of poultry: live attenuated *S. enterica*, sv. Typhimurium (Salmune^®^, CEVA Biomune) and a probiotic feed supplement composed of representatives of the LAB group: *Lactobacillus acidophilus*, *Lactobacillus casei*, *Enterococcus faecium*, and *Bifidobacterium bifidum* (PrimaLac^®^, Star Labs). Studies have shown that a single administration of the *Salmonella* strain and a daily intake of a probiotic dietary supplement change the microbiota of growing chickens. These differences, mainly related to changes in the amount of microorganisms forming microbiome, were sustained throughout the study. However, although the use of probiotics and bacterial vaccines affects the taxonomic composition of the microbiome, it has only a temporary or minor effect on the function and activity of the microbiome in non-stress growth conditions (Ballou et al., 2016). It cannot be ruled out that the main advantage of using probiotic strains in animal production is to enable a quick restoration of a disturbed or stunted microbiome to the normal functional state. Another advantage is that probiotics do not have a deleterious impact on microbial diversity. This is in stark contrast with antibiotics: it was found that the exposure of mice to antibiotics at an early age of life can induce deleterious changes in the microbiome that can persist even for several months after treatment (Nobel et al., 2015; Grazul et al., 2016).

Baldwin et al. also have checked how the administration of probiotics affects the composition of the microbiome. They assumed that the best opportunity to achieve a permanent colonization in birds and influence the development of microbiota is the early period of life, before the microbiome has established itself – in other words, that early inoculum may shape the gut microbiota of chickens for life. Therefore, they administered an inoculum of selected beneficial strains (*Lactobacillus ingluviei*, *Lactobacillus agilis*, and *Lactobacillus reuteri*), capable of persistently colonizing poultry, to one-day hatch chicks (single dose). The resulting changes in the probiotic group consisted of reduction in *Alistipes*- and *Ruminococcus*-related species (Baldwin et al., 2018). Probiotic treatment also increased *Bacteroides uniformis* species, which is known to have the potential to degrade the isoflavones in the gut and significantly improve metabolic and immunological dysfunction in mice with diet-induced obesity (Renouf and Hendrich, 2011; Gauffin Cano et al., 2012). The presence of the best-colonizing inoculated strain was higher in earlier days and then was reduced by day 28. So the probiotic inoculation had lasting effects on the development of the community rather than establishing dominance (Baldwin et al., 2018).

### LAB as Anti-*Campylobacter* Probiotics for Poultry

Applications of some of probiotic strains are also intended to reduce the numbers of pathogenic microorganisms in the gastrointestinal tract of chickens. Because a positive correlation between the number of *Campylobacter* in the chickens caeca and the number on carcasses was noted (Reich et al., 2008), any decrease of *Campylobacter* colonization level should lead to reduced contamination of the food chain. So far, many probiotic strains have been described that shown the ability to modulate intestinal microflora and the potential to reduce the number of enteropathogenic bacteria in poultry intestine. Research carried out prior to 2016 in relation to *Campylobacter* and *Salmonella* has been reviewed extensively by Saint-Cyr et al. (2016) and Gaggia et al. (2010).

A variety of bacteria (*Bacillus*, *Bifidobacterium*, *Enterococcus*, *Lactobacillus*, *Streptococcus*, and *Lactococcus* spp.) has been tested as probiotics in poultry (Lutful Kabir, 2009), but most of the studies focused on genus *Lactobacillus*, whose representatives belong to LAB group. *Lactobacillus* sp. naturally occur on raw food and feed materials, but it also natively resides in the chicken gastrointestinal tract. In chickens, treatment with various members of the *Lactobacillus* species has been shown to stimulate multiple aspects of the immune response (Lutful Kabir, 2009). Recently, Sefcova et al. conducted studies on the immune mechanisms underlying the intestinal response to *Campylobacter* infection in the presence of the probiotic *L. fermentum* CCM7514. The results indicate that the administration of *L. fermentum* to 4day old chickens exerts a positive effect on the intestinal architecture of birds exposed to pathogens and favorably regulates the expression of pro-inflammatory cytokines, which may lead to a more effective response to *Campylobacter* invasion. Unfortunately, no studies directly addressed the effect of *Lactobacillus* on the level of chicken intestine colonization by *Campylobacter* (Sefcova et al., 2020a,b).

*Lactobacillus* spp. constitute a diverse group of microorganisms in regard to their physiological attributes and genetic constitution. The strains are characterized not only by a varying ability to survive in the intestinal environment, but also by a type of interaction with epithelial surfaces and immune cells. The differences are noticeable even among strains of the same species; therefore, a careful consideration is needed during the selection of strains for probiotic preparations. It was even shown, albeit admittedly in mice, that different strains of the same species within phylum *Lactobacillus* may act in the opposite manner: *L. reuteri* L6798 was associated with weight gain, whereas *L. reuteri* ATCCPTA4659 was associated with weight loss (Fak and Backhed, 2012).

There are numerous reports of the antagonistic activity of *Lactobacillus* strains against *Campylobacter*, which is very often associated with the production of organic acids and/or bacteriocins. The ability of *Lactobacillus* to adhere to the intestinal epithelium is closely related to the concept of competitive exclusion (CE) and is also one of the most important selection criteria for probiotic strains. The adherence allows microorganisms to survive and temporarily colonize the digestive system, which is necessary to induce beneficial effects on the host, and is assessed by *in vitro* examination of aggregation, hydrophobicity of cell wall, and adhesion to extracellular proteins including intestinal mucus, fibronectin, and basement membrane matrix (Edelman et al., 2002; Gusils et al., 2003; Bouzaine et al., 2005; Rocha et al., 2012). For example, *Lactobacillus rhamnosus* LGG reduces adhesion efficacy of *C. jejuni* most significantly under co-culture conditions (Sikic Pogacar et al., 2020). Khaled Taha-Abdelaziz et al. have shown that five *Lactobacillus* spp. (*L. salivarius*, *L. johnsonii*, *L. reuteri*, *L. crispatus*, and *L. gasseri*) exhibited also an anti-*Campylobacter* activity *in vitro*. Organic acids produced by examined *Lactobacillus* strains lead to the destabilization of the *Campylobacter* cell wall and are responsible, at least partially, for inhibiting the pathogen growth. It was also found that the tested strains inhibited the production of the quorum sensing autoinducer-2 molecule by *C. jejun*i and decreased the expression of genes related to virulence, including the genes responsible for motility (*flaA*, *fl*aB, and *flhA*). Moreover, treatment of chickens’ macrophages with these lactobacilli enhances their phagocytic activity against *C. jejuni*. These results suggest that the administration of probiotic lactobacilli to chickens may not only reduce *C. jejuni* colonization, but may also impair *C. jejuni’s* ability to survive and invade intestinal epithelial cells (Taha-Abdelaziz et al., 2019).

There have been many studies showing the possibility of using LAB strains as anti-*Campylobacter* probiotics. However, much less research confirms the effectiveness of these preparations *in vivo*. Promising results were obtained by teams of M. Konkel and E. K. Jagusztyn-Krynicka. They observed that the administration of the *Lactobacillus* genus, i.e., *L. crispatus*, *L. salivarius*, *L. helveticus*, and *L. gallinarum*, to chickens leads to a reduction of the colonization level of bird cecum by *Campylobacter* (Neal-McKinney et al., 2012; Kobierecka et al., 2017). Mañes-Lázaro et al. described that *Lactobacillus johnsonii* FI9785 has the potential to control *C. jejuni* infection; however, it depends strictly on successful probiotic colonization (Manes-Lazaro et al., 2017). Nishiyama et al. showed that *Lactobacillus gasseri* SBT2055 suppressed *C. jejuni* colonization by c. 250-fold (Nishiyama et al., 2014) and identified a cell surface-associated aggregation-promoting factor APF1 as being important both for colonization of chickens and for reducing colonization by *C. jejuni in vivo* (Nishiyama et al., 2015). There is also a lot of research showing that the use of multispecies probiotics has a positive effect. Administration of a mixture of five strains (*L. salivarius*, *L. reuteri*, *E. faecium*, *Pediococcus acidilactici*, and *Bifidobacterium*) to chicken was effective in reducing *C. jejuni* colonization *in vivo*, giving a mean 6 log10 reduction compared with controls (Ghareeb et al., 2012). Addition of multispecies probiotic (Lavipan, JHJ, Poland) composed of *Lactococcus lactis, Carnobacterium divergens, L. casei, L. plantarum*, and *Saccharomyces cerevisiae* to a feed for broiler chickens was capable to reduce slightly the extent of *Campylobacter* spp. (Smialek et al., 2018). This is one of the few studies to date, that have evidenced a possible role of probiotics in preventing the shedding of *Campylobacter* spp. under field conditions, at the level of production (Smialek et al., 2018). Among the birds that received the probiotic, no *Campylobacter* was found in 25% of the intestinal samples and 100% of the pectoral muscles samples, while in the control group, growth of *Campylobacter* was observed in 100 and 50%, appropriately. The commonly used research facilities typically do not reflect field conditions, which include numerous on-farm sources of *Campylobacter* leading to possible recontamination of the flock during the rearing period. Thus the action of probiotic products should be validated through on-farm trials. The evaluation of the effectiveness in reducing *Campylobacter* of commercial feed additives was carried out by Mortada et al. It turned out that although the *in vitro* results indicated the effectiveness of the preparations used, *in vivo*, none of the treatments influenced the *Campylobacter* load in the cecum at the age of 42days (Mortada et al., 2020). The presented studies are summarized in Table 2.

**Table 2:** LAB as anti-*Campylobacter* probiotics for poultry.

LAB strain	Dose	Administration	Effect	Reference
*L. fermentum* CCM7514	∼10^9^CFU/0.2ml	daily for first 7days of life	- slight significant increase in weight – positive regulation of pro-inflammatory cytokine expression (upregulation of some type II cytokines (IL-4 and IL-13), downregulation of pro-inflammatory cytokines IL-15, IL-16, and interferon γ)	Sefcova et al., 2020a,b
*L. acidophilus*, NCFM *L. crispatus*, JCM 5810 *L. gallinarum*, ATCC 33199 *L. helveticus*, CNRZ32	∼10^8^CFU/0.5ml	1st and 4th day of life	reduction in *C. jejuni* colonization in broiler chickens	Neal-McKinney et al., 2012
*L. plantarum* PA18A and *L. plantarum* PA20A	∼10^8^CFU/0.1ml	1st and 4th day of life	slight reduction in *C. jejuni* colonization in broiler chickens	Kobierecka et al., 2017
*E. faecium*, *P. acidilactici*, *B. animalis*, *L. salivarius*, and *L. reuteri* (PoultryStar sol BIOMIN GmbH, Herzogenburg, Austria)	2mg/bird/day and 20mg/bird/day	from 1st day of life	significant reduction in colonization (there was no significant difference obtained between probiotic-treated groups)	Ghareeb et al., 2012
multispecies probiotic (Lavipan, JHJ, Poland) composed of *L. lactis* IBB500, *C. divergens* S-1, *L. casei* ŁOCK0915, *L. plantarum* ŁOCK0862, and *S. cerevisiae* ŁOCK 0141		0.05% probiotic in feed from first day of life	slight reduction in *Campylobacter* spp. colonization	Smialek et al., 2018
*L. gasseri* SBT2055 (LG2055)	∼10^8^CFU/0.1ml	daily for 14days after oral inoculation with *C. jejuni* 81176 (from 2nd day of life)	significant reduction in *C. jejuni* colonization	Nishiyama et al., 2014, 2015
*L. johnsonii* FI9785	∼10^8^CFU/0.1ml	1st and 8th day of life	reduction in *C. jejuni* colonization in chickens	Manes-Lazaro et al., 2017
PoultryStar ME (BIOMIN America, Inc.)+organic acids (OA): *L. reuteri, P. acidilactici, B. animalis*, and *E. faecium*		1st to 28th day of life – 0.05% probiotic in feed; 28th to 42nd day of life - 0.05% OA in feed	non-significant reduction in *C. coli* load in ceca	Mortada et al., 2020

Limiting *Campylobacter* in poultry production remains therefore a continuing challenge. Dissecting complex interactions between *Campylobacter* and the GIT resident microbial community as well as immune system of the bird appear to be a necessary step facilitating development of an effective probiotic preparation.

Probiotics combined with prebiotics form synbiotics. Prebiotics are a non-viable food component that confer health benefit(s) on the host associated with modulation of the microbiota. In the last decade, the use of synbiotic supplements in poultry flocks has been intensively investigated. The prebiotics applicated most often were galacto-oligosaccharides (GOS), fructo-oligosaccharides, or xylooligosaccharides (XOS). Baffoni et al. showed that *B. longum* PCB133 associated with XOS is effective in reducing *C. jejuni* colonization. Moreover, it turned out that the administration of this synbiotic at the beginning of animal life when the gut microbiota is still under development and more susceptible to changes is more effective (Baffoni et al., 2017). Last presented study showed that dietary supplementation with the prebiotic GOS affects the number of autochthonous synbiotic species in the intestines, precisely *L. johnsonii*. This is linked to improved performance and the expression of cytokines and chemokines significant to prime innate intestinal immune systems (Richards et al., 2020).

### Lactic Acid Bacteria as Vaccine Vehicle

Due to its beneficial properties, LAB is common components in commercial probiotics used in poultry agriculture. Recently, an increasing attention has been paid to their new potential biomedical application as a live oral vaccine delivery platform (live vaccine vector). In this respect, the most promising research is carried out on *L. lactis* and several species of the genus *Lactobacillus: L. rhamnosus, L. casei, L. bulgaricus, L. salivarius, L. plantarum, L. acidophilus, L. helveticus, L. gasseri*, and *Streptococcus gordonii*.

In recent years, several studies have explored the use of genetically modified *L. lactis* and some *Lactobacillus* spp. to express conserved *Campylobacter* antigens. The use of these species has its justification. *L. lactis* is able to survive in the digestive tract of humans and animals and to establish a transient colonization of the intestine through adhesion to mucus layer (Wang et al., 2011), while some *Lactobacillus* strains colonize broiler chicks more persistently and at a higher level (Spivey et al., 2014).

**Figure 3:**
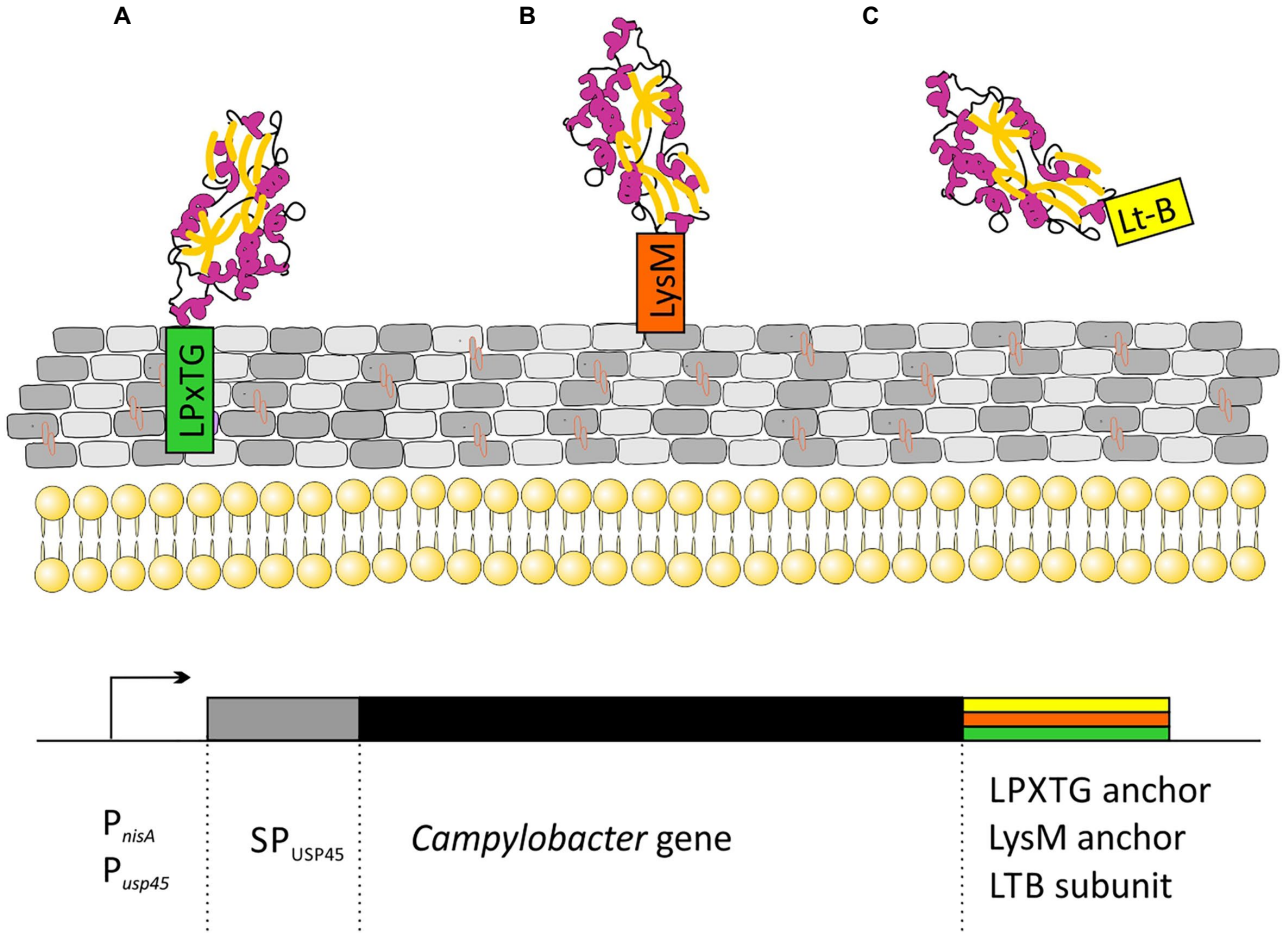
Schematic representation of strategies for the cloning of *C. jejuni* genes for secretory expression in *Lactococcus lactis:* **(A)** *Campylobacter* protein covalently attached to peptidoglycan *via* the CWA domain from M6 or YndF proteins; **(B)** *Campylobacter* protein non-covalently attached to peptidoglycan *via* LysM domains from AcmA protein; and **(C)** secreted *Campylobacter* protein fused to labile enterotoxin subunit B domain. Transcription of the cloned genes is driven by an nisin-inducible P_nisA_ promoter or strong, constitutive P_usp45_ promoter. SP_usp45_ – signal peptide of Usp45, the major Sec-dependent protein secreted by *L. lactis*.

All the strategies proposed so far are based on lactic acid bacteria strains displaying different *C. jejuni* antigens on their surface. Many types of proteins are attached to the cell walls of Gram-positive bacteria. Among them are: (i) proteins specifically recognized by sortase and covalently linked to peptidoglycan through an LPxTG motif; (ii) proteins attached in a non-covalent manner through specific protein domains (LysM or SH3 domains); (iii) membrane anchored (lipoprotein); and (iv) proteins having one or more transmembrane domains. The first two strategies for attaching proteins to peptidoglycan are the most popular in vaccine development (Figure 3).

In Kobierecka et al. (2016a) used *L. lactis* strain presenting two *Campylobacter* antigens on the cell surface. Using C-terminus of the *L. lactis* YndF containing the LPTXG motif, they decorated the cell wall with *Campylobacter* rCjaAD hybrid protein composed of CjaA antigen presenting CjaD peptide epitopes (Kobierecka et al., 2016a). CjaA is a glycosylated, strongly immunogenic lipoprotein anchored in the inner membrane of the *Campylobacter* cell. It is a component of the ABC transport system with cysteine as its natural ligand (Muller et al., 2005; Wyszynska et al., 2008). While CjaD is peptidoglycan-associated protein (Pal), a part of the Tol-Pal system which is important for maintaining outer membrane integrity.

The same group also investigated the effectiveness of non-live carrier of *Campylobacter* antigens: *Lactobacillus salivarius* GEM particles (Gram-positive enhancer matrix particles which were obtained by chemical pre-treatment of bacterial cells with trichloroacetic acid). GEM particles presented the same two antigens: CjaA and CjaD on their surface but this time, the antigens were fused with the protein anchor (PA) of the *L. lactis* peptidoglycan hydrolase AcmA, which comprises 3 LysM motifs non-covalently bind to peptidoglycan (Kobierecka et al., 2015).

Two more research groups presented a similar strategy. In 2019, Gorain and colleagues used *L. lactis* strain as a vector and the *C. jejuni* adhesin, JlpA as the antigen. JlpA is a lipoprotein that interacts with intestinal heat shock protein (Hsp90α) and is involved in triggering a pro-inflammatory response. They linked the JlpA protein to the *L. lactis* protease USP4 signal peptide at the N-terminus and to the M6 protein cell wall anchor motif (with LPxTG sequence) of *Streptococcus pyogenes* (CWA_M6) at the C-terminus. The presence of the CWA_M6 motif ensured the localization of the JlpA protein on the surface of the bacterial cells. Additionally, it has been proven that the JlpA protein anchored in the peptidoglycan of *L. lactis* cells retains its biological activity (Gorain et al., 2020).

Wang’s group proposed yet another method, in which the *C. jejuni* CjaA antigen was not anchored to the peptidoglycan, but was secreted from the cell. CjaA was coexpressed with the *usp45* signal peptide supporting secretory expression and with the *E. coli* heat labile enterotoxin subunit B, which acted as a mucosal adjuvant (Newsted et al., 2015; Sun et al., 2017; Wang et al., 2020).

All presented strategies were successful and *L. lactis* cells produced sufficient *C. jejuni* antigens to elicit an immune response in the vaccinated animals. It must be admitted, however, that the protective effect against colonization with *Campylobacter* was not impressive. Chickens immunized with *L. lactis*, producing surface exposed hybrid protein rCjaAD, showed moderate 1 log10 reduction of *Campylobacter* load in the caecum compared to the control group. The vaccine consisting of *L. salivarius* GEM particles presenting on the surface CjaA and CjaD did not produce any protective effect, but GEM *L. salivarius* particles presenting hybrid rCjaAD administered *in ovo* to the chicken embryos slightly lowered the level of intestine colonization by *Campylobacter* in comparison with the control group (the median reduction of *C. jejuni* cecal contents was 1 log10 for *in ovo* immunization with GEM particles containing rCjaAD; Kobierecka et al., 2016). A similar effect was obtained when the animals were orally immunized with *L. lactis* expressing the JlpA. Significant reduction in the bacterial load, 7days post-challenge, was observed in the animals immunized with *L. lactis* expressing JlpA as well as in the group which was administered subcutaneously with IFA-JlpA antigen (purified JlpA protein emulsified in incomplete Freund’s adjuvant) compared to unimmunized birds in the control group. The reduction in *C. jejuni* colonization demonstrated by Gorain’s team was at the same level as the effect described by Kobierecka et al. and amounted ~1 log10 (Kobierecka et al., 2015; Gorain et al., 2020). The immunization of chickens with the *L. lactis* strain secreting the CjaALtB protein showed no significant protective effect. Initially, 5days post-challenge, Wang et al. observed a significant reduction of cecal *C. jejuni* (by 2.35 log10), but 9days post-challenge, all bird groups were colonized at the same level (Wang et al., 2020). Despite these unsatisfactory results, all research groups showed that the intragastric administration of *L. lactis* cells expressing *Campylobacter* proteins induced adaptive immune responses in chickens. In the most comprehensive study, Gorain et al. demonstrated significant rise in IgA level in the vaccinated animals compared to the control groups of birds. They also proved that *L. lactis* expressing JlpA protein activated an innate immune response by triggering TLR-2 intracellular signaling cascade (Gorain et al., 2020).

These results show that LAB, in particular *L. lactis*, can be an effective platform for the delivery of *Campylobacter* antigens to the immune system of birds, as previously demonstrated with other pathogens, e.g., Avian Influenza Virus *Clostridium tetani* toxin, *Brucella abortus, Rhodococcus equi*, and *Streptococcus pneumoniae* (pneumococcus; Wells et al., 1993; Medina et al., 2010; Cauchard et al., 2011; Saez et al., 2012; Lahiri et al., 2019).

It should be also emphasized that *L. lactis*-based vaccines are capable of activating both types of the immune response and inducing a specific mucosal response, but the problem of how to enforce the immune response of chickens to get a better protective effect against *Campylobacter* infection is still awaiting a solution. Therefore, it seems that exploring new *Campylobacter* antigens and searching for effective adjuvants are still important directions for the further scientific development in this field.

## Conclusion

It is estimated that over the next 20years, chicken production will have to increase 4-fold to satisfy the growing global demand. Therefore, the sustainable production of safe meat, not only chicken meat, is an international priority. The key question is whether it can be done in a way that does not increase the risk to public health and at the same time protects the health and welfare of animals.

In recent years, there have been more and more reports on taxonomic differences and the development of microbial communities in the gut of farm animals, in particular chickens. The development of affordable next-generation DNA sequencing techniques has made it possible to study the diversity of this important ecosystem in detail and to link changes in microbiota to animal health. Understanding the relationship between microorganisms forming farm animal microbiome might certainly help to develop alternative strategies for replacing antibiotics in modern poultry production and ensuring food safety.

There are high hopes for probiotics at the moment. Subtle manipulations in the composition of the microflora of the gastrointestinal tract, resulting from the use of probiotics, have beneficial effects on maintaining health through diversity, stability of metabolites, and modulation of the immune system. Probiotics modulate the environment of gastrointestinal tract, act synergistically with the immune system host, reduce the risk of digestive tract diseases, and for these reasons they can find applications in animal husbandry. Without a doubt, a thorough understanding of the normal succession in the gut microbiota can aid the development and optimization of the probiotic-based strategies.

The intention of this paper was to review the current knowledge regarding the ability of probiotic strains to eliminate or reduce the load of dangerous human pathogens in the animal intestinal tract. Excessive growth of pathogenic bacteria in the digestive system is often a result of disordered composition of the intestinal microbiome. The flagship example is an extremely dangerous *Clostridium difficile* infection in humans subjected to antibiotic therapy. We now know more and more about mutual dependencies between microorganisms. Typical cases include (i) a presence of one microorganism generates a niche for other, pathogenic microorganisms to colonize the host, (ii) one microorganism predisposes the host to be colonized by other microorganisms, and (iii) two or more nonpathogenic microorganisms together cause disease. The gut microbiome is currently considered as a “super organ” involved in a range of disease states. If we learn to heal this super organ, we will get a tool to control some diseases.

Probiotics are one of the possible treatments that demonstrated potential to reduce the intestinal colonization by pathogens. However, their beneficial effect is largely dependent on the type and amount of probiotic bacterial strains used, as well as their dose, method and time of administration. Also, the effects on feed intake, microbial fermentation, and intestinal architecture showed a differential pattern between challenged and non-challenged animals. This strongly indicates that there is still a need for further searches for new strains or new combinations of known probiotic strains.

## Author Contributions

AW and RG were responsible for the manuscript writing. All authors contributed to the article and approved the submitted version.

## Funding

This work was supported by the grants from the National Science Center, Poland (grant no 2016/21/B/NZ6/01141).

## Conflict of Interest

The authors declare that the research was conducted in the absence of any commercial or financial relationships that could be construed as a potential conflict of interest.

## Publisher’s Note

All claims expressed in this article are solely those of the authors and do not necessarily represent those of their affiliated organizations, or those of the publisher, the editors and the reviewers. Any product that may be evaluated in this article, or claim that may be made by its manufacturer, is not guaranteed or endorsed by the publisher.

## Footnotes

^1^ https://www.who.int

## References

[ref1] Abt M. C. Artis D. (2009). The intestinal microbiota in health and disease: the influence of microbial products on immune cell homeostasis. Curr. Opin. Gastroenterol. 25, 496–502. doi: 10.1097/MOG.0b013e328331b6b4, PMID: 19770652 PMC4737592

[ref2] Andersson D. I. Hughes D. (2014). Microbiological effects of sublethal levels of antibiotics. Nat. Rev. Microbiol. 12, 465–478. doi: 10.1038/nrmicro3270, PMID: 24861036

[ref3] Apajalahti J. Kettunen A. Graham H. (2004). Characteristics of the gastrointestinal microbial communities, with special reference to the chicken. World’s Poultry Sci. J. 60, 223–232. doi: 10.1079/WPS20040017

[ref4] Arsi K. Donoghue A. M. Woo-Ming A. Blore P. J. Donoghue D. J. (2015). The efficacy of selected probiotic and prebiotic combinations in reducing *campylobacter* colonization in broiler chickens. J. Appl. Poult. Res. 24, 327–334. doi: 10.3382/japr/pfv032 25581198

[ref5] Atarashi K. Honda K. (2011). Microbiota in autoimmunity and tolerance. Curr. Opin. Immunol. 23, 761–768. doi: 10.1016/j.coi.2011.11.002, PMID: 22115876

[ref6] Awad W. A. Dublecz F. Hess C. Dublecz K. Khayal B. Aschenbach J. R.. (2016). *Campylobacter jejuni* colonization promotes the translocation of *Escherichia coli* to extra-intestinal organs and disturbs the short-chain fatty acids profiles in the chicken gut. Poult. Sci. 95, 2259–2265. doi: 10.3382/ps/pew151, PMID: 27143773

[ref7] Awad W. A. Hess C. Hess M. (2018). Re-thinking the chicken-*Campylobacter jejuni* interaction: a review. Avian Pathol. 47, 352–363. doi: 10.1080/03079457.2018.1475724, PMID: 29764197

[ref8] Awad W. A. Molnar A. Aschenbach J. R. Ghareeb K. Khayal B. Hess C.. (2015). *Campylobacter* infection in chickens modulates the intestinal epithelial barrier function. Innate Immun. 21, 151–160. doi: 10.1177/1753425914521648, PMID: 24553586

[ref9] Baffoni L. Gaggia F. Garofolo G. Di Serafino G. Buglione E. Di Giannatale E.. (2017). Evidence of *Campylobacter jejuni* reduction in broilers with early synbiotic administration. Int. J. Food Microbiol. 251, 41–47. doi: 10.1016/j.ijfoodmicro.2017.04.001, PMID: 28390936

[ref10] Baldwin S. Hughes R. J. Hao Van T. T. Moore R. J. Stanley D. (2018). At-hatch administration of probiotic to chickens can introduce beneficial changes in gut microbiota. PLoS One 13:e0194825. doi: 10.1371/journal.pone.0194825, PMID: 29570728 PMC5865720

[ref11] Ballou A. L. Ali R. A. Mendoza M. A. Ellis J. C. Hassan H. M. Croom W. J.. (2016). Development of the Chick Microbiome: How Early Exposure Influences Future Microbial Diversity. Front. Vet. Sci. 3:2. doi: 10.3389/fvets.2016.00002, PMID: 26835461 PMC4718982

[ref12] Baurhoo B. Ferket P. R. Zhao X. (2009). Effects of diets containing different concentrations of mannanoligosaccharide or antibiotics on growth performance, intestinal development, cecal and litter microbial populations, and carcass parameters of broilers. Poult. Sci. 88, 2262–2272. doi: 10.3382/ps.2008-00562, PMID: 19834074

[ref13] Bermudez-Brito M. Plaza-Diaz J. Munoz-Quezada S. Gomez-Llorente C. Gil A. (2012). Probiotic mechanisms of action. Ann. Nutr. Metab. 61, 160–174. doi: 10.1159/000342079, PMID: 23037511

[ref14] Borda-Molina D. Seifert J. Camarinha S. A. (2018). Current Perspectives of the Chicken Gastrointestinal Tract and Its Microbiome. Computat. Struct. Biotechnol. J. 16, 131–139. doi: 10.1016/j.csbj.2018.03.002, PMID: 30026889 PMC6047366

[ref15] Borda-Molina D. Vital M. Sommerfeld V. Rodehutscord M. Camarinha-Silva A. (2016). Insights into Broilers' Gut Microbiota Fed with Phosphorus, Calcium, and Phytase Supplemented Diets. Front. Microbiol. 7:2033. doi: 10.3389/fmicb.2016.02033, PMID: 28066358 PMC5165256

[ref16] Bouzaine T. Dauphin R. D. Thonart P. Urdaci M. C. Hamdi M. (2005). Adherence and colonization properties of *Lactobacillus rhamnosus* TB1, a broiler chicken isolate. Lett. Appl. Microbiol. 40, 391–396. doi: 10.1111/j.1472-765X.2005.01684.x, PMID: 15836745

[ref17] Boyd Y. Herbert E. G. Marston K. L. Jones M. A. Barrow P. A. (2005). Host genes affect intestinal colonisation of newly hatched chickens by *Campylobacter jejuni*. Immunogenetics 57, 248–253. doi: 10.1007/s00251-005-0790-6, PMID: 15900496

[ref18] Buckley A. M. Wang J. Hudson D. L. Grant A. J. Jones M. A. Maskell D. J.. (2010). Evaluation of live-attenuated *Salmonella* vaccines expressing *Campylobacter* antigens for control of *C. jejuni* in poultry. Vaccine 28, 1094–1105. doi: 10.1016/j.vaccine.2009.10.018, PMID: 19853682

[ref19] Byrd J. A. Hargis B. M. Caldwell D. J. Bailey R. H. Herron K. L. McReynolds J. L.. (2001). Effect of lactic acid administration in the drinking water during preslaughter feed withdrawal on *Salmonella* and *Campylobacter* contamination of broilers. Poult. Sci. 80, 278–283. doi: 10.1093/ps/80.3.278, PMID: 11261556

[ref20] Cauchard S. Bermudez-Humaran L. G. Blugeon S. Laugier C. Langella P. Cauchard J. (2011). Mucosal co-immunization of mice with recombinant lactococci secreting VapA antigen and leptin elicits a protective immune response against *Rhodococcus equi* infection. Vaccine 30, 95–102. doi: 10.1016/j.vaccine.2011.10.026, PMID: 22019740

[ref21] Cawthraw S. A. Newell D. G. (2010). Investigation of the presence and protective effects of maternal antibodies against *Campylobacter jejuni* in chickens. Avian Dis. 54, 86–93. doi: 10.1637/9004-072709-Reg.1, PMID: 20408404

[ref22] Chaveerach P. Keuzenkamp D. A. Urlings H. A. Lipman L. J. van Knapen F. (2002). In vitro study on the effect of organic acids on *Campylobacter jejuni/coli* populations in mixtures of water and feed. Poult. Sci. 81, 621–628. doi: 10.1093/ps/81.5.621, PMID: 12033410

[ref23] Clavijo V. Florez M. J. V. (2018). The gastrointestinal microbiome and its association with the control of pathogens in broiler chicken production: a review. Poult. Sci. 97, 1006–1021. doi: 10.3382/ps/pex359, PMID: 29253263 PMC5850219

[ref24] Conlan A. J. Coward C. Grant A. J. Maskell D. J. Gog J. R. (2007). *Campylobacter jejuni* colonization and transmission in broiler chickens: a modelling perspective. J. R. Soc. Interface 4, 819–829. doi: 10.1098/rsif.2007.1015, PMID: 17472905 PMC2077357

[ref25] Conlan A. J. Line J. E. Hiett K. Coward C. Van Diemen P. M. Stevens M. P.. (2011). Transmission and dose-response experiments for social animals: a reappraisal of the colonization biology of *Campylobacter jejuni* in chickens. J. R. Soc. Interface 8, 1720–1735. doi: 10.1098/rsif.2011.0125, PMID: 21593028 PMC3203482

[ref26] Connerton P. L. Richards P. J. Lafontaine G. M. O'Kane P. M. Ghaffar N. Cummings N. J.. (2018). The effect of the timing of exposure to *Campylobacter jejuni* on the gut microbiome and inflammatory responses of broiler chickens. Microbiome 6:88. doi: 10.1186/s40168-018-0477-5, PMID: 29753324 PMC5948730

[ref27] Costa M. C. Stampfli H. R. Arroyo L. G. Allen-Vercoe E. Gomes R. G. Weese J. S. (2015). Changes in the equine fecal microbiota associated with the use of systemic antimicrobial drugs. BMC Vet. Res. 11:19. doi: 10.1186/s12917-015-0335-7, PMID: 25644524 PMC4323147

[ref28] Cressman M. D. Yu Z. Nelson M. C. Moeller S. J. Lilburn M. S. Zerby H. N. (2010). Interrelations between the microbiotas in the litter and in the intestines of commercial broiler chickens. Appl. Environ. Microbiol. 76, 6572–6582. doi: 10.1128/AEM.00180-10, PMID: 20693454 PMC2950482

[ref29] Dasti J. I. Tareen A. M. Lugert R. Zautner A. E. Gross U. (2010). *Campylobacter jejuni*: a brief overview on pathogenicity-associated factors and disease-mediating mechanisms. Int. J. Med. Microbiol. 300, 205–211. doi: 10.1016/j.ijmm.2009.07.002, PMID: 19665925

[ref30] De Cesare A. Sirri F. Manfreda G. Moniaci P. Giardini A. Zampiga M.. (2017). Effect of dietary supplementation with *Lactobacillus acidophilus* D2/CSL (CECT 4529) on caecum microbioma and productive performance in broiler chickens. PLoS One 12:e0176309. doi: 10.1371/journal.pone.0176309, PMID: 28472118 PMC5417446

[ref31] De Los Santos F. Donoghue A. M. Venkitanarayanan K. Metcalf J. H. Reyes-Herrera I. Dirain M. L.. (2009). The natural feed additive caprylic acid decreases *Campylobacter jejuni* colonization in market-aged broiler chickens. Poult. Sci. 88, 61–64. doi: 10.3382/ps.2008-00228, PMID: 19096058

[ref32] Dibner J. J. Richards J. D. (2005). Antibiotic growth promoters in agriculture: history and mode of action. Poult. Sci. 84, 634–643. doi: 10.1093/ps/84.4.634, PMID: 15844822

[ref33] Duncan S. H. Holtrop G. Lobley G. E. Calder A. G. Stewart C. S. Flint H. J. (2004). Contribution of acetate to butyrate formation by human faecal bacteria. Br. J. Nutr. 91, 915–923. doi: 10.1079/BJN20041150, PMID: 15182395

[ref34] Duquenoy A. Ania M. Boucher N. Reynier F. Boucinha L. Andreoni C.. (2020). Caecal microbiota compositions from 7-day-old chicks reared in high-performance and low-performance industrial farms and systematic culturomics to select strains with anti-*Campylobacter* activity. PLoS One 15:e0237541. doi: 10.1371/journal.pone.0237541, PMID: 32834007 PMC7446796

[ref35] Edelman S. Westerlund-Wikstrom B. Leskela S. Kettunen H. Rautonen N. Apajalahti J.. (2002). *In vitro* adhesion specificity of indigenous *Lactobacilli* within the avian intestinal tract. Appl. Environ. Microbiol. 68, 5155–5159. doi: 10.1128/AEM.68.10.5155-5159.2002, PMID: 12324367 PMC126384

[ref36] EFSA (2016). The European Union summary report on trends and sources of zoonoses, zoonotic agents and food-borne outbreaks in 2015. EFSA J. 14:e04634. doi: 10.2903/j.efsa.2016.s0512, PMID: 32625371 PMC7009962

[ref37] EFSA and ECDC (2017). The European Union summary report on trends and sources of zoonoses, zoonotic agents and food-borne outbreaks in 2016. EFSA J. 15:228. doi: 10.2903/j.efsa.2017.5077, PMID: 32625371 PMC7009962

[ref38] EFSA and ECDC (2021). The European Union One Health 2019 Zoonoses Report. EFSA J. 19:286. doi: 10.2903/j.efsa.2021.6406, PMID: 33680134 PMC7913300

[ref39] EFSA Panel on Biological Hazards (BIOHAZ) (2011). Scientific opinion on *Campylobacter* in broiler meat production: control options and performance objectives and/or targets at different stages of the food chain. EFSA J. 9:2105. doi: 10.2903/j.efsa.2011.2190, PMID: 32313582 PMC7163696

[ref40] EMA (2017). Sales of veterinary antimicrobial agents in 30 European countries in 2015. *European Surveillance of Veterinary Antimicrobial Consumption* EMA/184855/2017.

[ref41] EMA (2020). Sales of veterinary antimicrobial agents in 31 European countries in 2018. *European Surveillance of Veterinary Antimicrobial Consumption* EMA/24309/2020.

[ref42] Fak F. Backhed F. (2012). *Lactobacillus reuteri* prevents diet-induced obesity, but not atherosclerosis, in a strain dependent fashion in Apoe−/− mice. PLoS One 7:e46837. doi: 10.1371/journal.pone.0046837, PMID: 23056479 PMC3467285

[ref43] FAO-WHO (2006). Probiotics in food. health and nutritional properties and guidelines for evaluation. *FAO Food Nutr Pap* 85. doi:ISBN: 92-5-105513-0

[ref44] FDA (2015). Federal Register, Vol. 80, No. 106, June 3, 2015, Docket No. FDA–2010–N–0155, RIN0910–AG95.

[ref45] FDA (2019). Summary report On antimicrobials sold or distributed for use in food-producing animals. Available at: https://www.fda.gov/animal-veterinary/cvm-updates/fda-releases-annual-summary-report-antimicrobials-sold-or-distributed-2019-use-food-producing

[ref46] Gaggia F. Mattarelli P. Biavati B. (2010). Probiotics and prebiotics in animal feeding for safe food production. Int. J. Food Microbiol. 141:(Suppl. 1), S15–S28. doi: 10.1016/j.ijfoodmicro.2010.02.031, PMID: 20382438

[ref47] Gauffin Cano P. Santacruz A. Moya A. Sanz Y. (2012). Bacteroides uniformis CECT 7771 ameliorates metabolic and immunological dysfunction in mice with high-fat-diet induced obesity. PLoS One 7:e41079. doi: 10.1371/journal.pone.0041079, PMID: 22844426 PMC3406031

[ref48] Ghareeb K. Awad W. A. Mohnl M. Porta R. Biarnes M. Bohm J.. (2012). Evaluating the efficacy of an avian-specific probiotic to reduce the colonization of *Campylobacter jejuni* in broiler chickens. Poult. Sci. 91, 1825–1832. doi: 10.3382/ps.2012-02168, PMID: 22802174

[ref49] Goodfellow J. A. Willison H. J. (2016). Guillain-Barre syndrome: a century of progress. Nat. Rev. Neurol. 12, 723–731. doi: 10.1038/nrneurol.2016.172, PMID: 27857121

[ref50] Gorain C. Singh A. Bhattacharyya S. Kundu A. Lahiri A. Gupta S.. (2020). Mucosal delivery of live *Lactococcus lactis* expressing functionally active JlpA antigen induces potent local immune response and prevent enteric colonization of *Campylobacter jejuni* in chickens. Vaccine 38, 1630–1642. doi: 10.1016/j.vaccine.2019.12.064, PMID: 31932136

[ref51] Grazul H. Kanda L. L. Gondek D. (2016). Impact of probiotic supplements on microbiome diversity following antibiotic treatment of mice. Gut Microbes 7, 101–114. doi: 10.1080/19490976.2016.1138197, PMID: 26963277 PMC4856465

[ref52] Gusils C. Oppezzo O. Pizarro R. Gonzalez S. (2003). Adhesion of probiotic lactobacilli to chick intestinal mucus. Can. J. Microbiol. 49, 472–478. doi: 10.1139/w03-055, PMID: 14569288

[ref53] Guyard-Nicodeme M. Keita A. Quesne S. Amelot M. Poezevara T. Le Berre B.. (2016). Efficacy of feed additives against *Campylobacter* in live broilers during the entire rearing period. Poult. Sci. 95, 298–305. doi: 10.3382/ps/pev303, PMID: 26706356

[ref54] Haghighi H. R. Abdul-Careem M. F. Dara R. A. Chambers J. R. Sharif S. (2008). Cytokine gene expression in chicken cecal tonsils following treatment with probiotics and *salmonella* infection. Vet. Microbiol. 126, 225–233. doi: 10.1016/j.vetmic.2007.06.026, PMID: 17681719

[ref55] Hald B. Sommer H. M. Skovgard H. (2007). Use of fly screens to reduce *campylobacter* spp. introduction in broiler houses. Emerg. Infect. Dis. 13, 1951–1953. doi: 10.3201/eid1312.070488, PMID: 18258057 PMC2876755

[ref56] Han Z. Pielsticker C. Gerzova L. Rychlik I. Rautenschlein S. (2016). The influence of age on *campylobacter jejuni* infection in chicken. Dev. Comp. Immunol. 62, 58–71. doi: 10.1016/j.dci.2016.04.020, PMID: 27131855

[ref57] Havelaar A. H. Ivarsson S. Lofdahl M. Nauta M. J. (2013). Estimating the true incidence of campylobacteriosis and salmonellosis in the European Union, 2009. Epidemiol. Infect. 141, 293–302. doi: 10.1017/S0950268812000568, PMID: 22717051 PMC9152072

[ref58] He T. Long S. Mahfuz S. Wu D. Wang X. Wei X.. (2019). Effects of Probiotics as Antibiotics Substitutes on Growth Performance, Serum Biochemical Parameters, Intestinal Morphology, and Barrier Function of Broilers. Animals 9:985. doi: 10.3390/ani9110985, PMID: 31752114 PMC6912548

[ref59] Hermans D. Pasmans F. Heyndrickx M. Van Immerseel F. Martel A. Van Deun K.. (2012a). A tolerogenic mucosal immune response leads to persistent *Campylobacter jejuni* colonization in the chicken gut. Crit. Rev. Microbiol. 38, 17–29. doi: 10.3109/1040841X.2011.615298, PMID: 21995731

[ref60] Hermans D. Pasmans F. Messens W. Martel A. Van Immerseel F. Rasschaert G.. (2012b). Poultry as a host for the zoonotic pathogen *Campylobacter jejuni*. Vector Borne Zoonotic Dis. 12, 89–98. doi: 10.1089/vbz.2011.0676, PMID: 22133236

[ref61] Hermans D. Van Steendam K. Verbrugghe E. Verlinden M. Martel A. Seliwiorstow T.. (2014). Passive immunization to reduce *Campylobacter jejuni* colonization and transmission in broiler chickens. Vet. Res. 45:27. doi: 10.1186/1297-9716-45-27, PMID: 24589217 PMC3996517

[ref62] Hilmarsson H. Thormar H. Thrainsson J. H. Gunnarsson E. Dadadottir S. (2006). Effect of glycerol monocaprate (monocaprin) on broiler chickens: an attempt at reducing intestinal *Campylobacter* infection. Poult. Sci. 85, 588–592. doi: 10.1093/ps/85.4.588, PMID: 16615341

[ref63] Hughes L. Hermans P. Morgan K. (2008). Risk factors for the use of prescription antibiotics on UK broiler farms. J. Antimicrob. Chemother. 61, 947–952. doi: 10.1093/jac/dkn017, PMID: 18227086

[ref64] Humphrey S. Chaloner G. Kemmett K. Davidson N. Williams N. Kipar A.. (2014). *Campylobacter jejuni* is not merely a commensal in commercial broiler chickens and affects bird welfare. mBio 5:e01364-14. doi: 10.1128/mBio.01364-14, PMID: 24987092 PMC4161246

[ref65] Ijaz U. Z. Sivaloganathan L. Mckenna A. Richmond A. Kelly C. Linton M.. (2018). Comprehensive longitudinal microbiome analysis of the chicken cecum reveals a shift from competitive to environmental drivers and a window of opportunity for *Campylobacter*. Front. Microbiol. 9:2452. doi: 10.3389/fmicb.2018.02452, PMID: 30374341 PMC6196313

[ref66] Johnson T. J. Youmans B. P. Noll S. Cardona C. Evans N. P. Karnezos T. P.. (2018). A Consistent and Predictable Commercial Broiler Chicken Bacterial Microbiota in Antibiotic-Free Production Displays Strong Correlations with Performance. Appl. Environ. Microbiol. 84:e00362-18. doi: 10.1128/AEM.00362-18, PMID: 29625981 PMC5981067

[ref67] Kaakoush N. O. Castano-Rodriguez N. Mitchell H. M. Man S. M. (2015). Global Epidemiology of *Campylobacter* Infection. Clin. Microbiol. Rev. 28, 687–720. doi: 10.1128/CMR.00006-15, PMID: 26062576 PMC4462680

[ref68] Kaakoush N. O. Sodhi N. Chenu J. W. Cox J. M. Riordan S. M. Mitchell H. M. (2014). The interplay between *Campylobacter* and *Helicobacter* species and other gastrointestinal microbiota of commercial broiler chickens. Gut Pathog. 6:18. doi: 10.1186/1757-4749-6-18, PMID: 24940386 PMC4060860

[ref69] Kaiser P. Howell M. M. Fife M. Sadeyen J. R. Salmon N. Rothwell L.. (2009). Towards the selection of chickens resistant to *Salmonella* and *Campylobacter* infections. Bull. Mem. Acad. R. Med. Belg. 164, 17–25. PMID: 19718951

[ref70] Kalischuk L. D. Leggett F. Inglis G. D. (2010). *Campylobacter jejuni* induces transcytosis of commensal bacteria across the intestinal epithelium through M-like cells. Gut Pathog. 2:14. doi: 10.1186/1757-4749-2-14, PMID: 21040540 PMC2987776

[ref71] Khan S. Moore R. J. Stanley D. Chousalkar K. K. (2020). The Gut Microbiota of Laying Hens and Its Manipulation with Prebiotics and Probiotics To Enhance Gut Health and Food Safety. Appl. Environ. Microbiol. 86:e00600-20. doi: 10.1128/AEM.00600-20, PMID: 32332137 PMC7301851

[ref72] Kim S. A. Jang M. J. Kim S. Y. Yang Y. Pavlidis H. O. Ricke S. C. (2019). Potential for Prebiotics as Feed Additives to Limit Foodborne *Campylobacter* Establishment in the Poultry Gastrointestinal Tract. Front. Microbiol. 10:91. doi: 10.3389/fmicb.2019.00091, PMID: 30804900 PMC6371025

[ref73] Kittler S. Fischer S. Abdulmawjood A. Glunder G. Klein G. (2013). Effect of bacteriophage application on *Campylobacter jejuni* loads in commercial broiler flocks. Appl. Environ. Microbiol. 79, 7525–7533. doi: 10.1128/AEM.02703-13, PMID: 24077703 PMC3837725

[ref74] Kobierecka P. A. Olech B. Ksiazek M. Derlatka K. Adamska I. Majewski P. M.. (2016a). Cell Wall Anchoring of the *Campylobacter* Antigens to *Lactococcus lactis*. Front. Microbiol. 7:165. doi: 10.3389/fmicb.2016.00165, PMID: 26925040 PMC4757695

[ref75] Kobierecka P. A. Wyszynska A. K. Aleksandrzak-Piekarczyk T. Kuczkowski M. Tuzimek A. Piotrowska W.. (2017). *In vitro* characteristics of *Lactobacillus* spp. strains isolated from the chicken digestive tract and their role in the inhibition of *Campylobacter* colonization. MicrobiologyOpen 6:e00512. doi: 10.1002/mbo3.512, PMID: 28736979 PMC5635155

[ref76] Kobierecka P. A. Wyszynska A. K. Gubernator J. Kuczkowski M. Wisniewski O. Maruszewska M.. (2016b). Chicken Anti-*Campylobacter* Vaccine - Comparison of Various Carriers and Routes of Immunization. Front. Microbiol. 7:740. doi: 10.3389/fmicb.2016.00740, PMID: 27242755 PMC4872485

[ref77] Kobierecka P. Wyszynska A. Maruszewska M. Wojtania A. Zylinska J. Bardowski J.. (2015). Lactic acid bacteria as a surface display platform for *Campylobacter jejuni* antigens. J. Mol. Microbiol. Biotechnol. 25, 1–10. doi: 10.1159/000368780, PMID: 25662187

[ref78] Kumar S. Chen C. Indugu N. Werlang G. O. Singh M. Kim W. K.. (2018). Effect of antibiotic withdrawal in feed on chicken gut microbial dynamics, immunity, growth performance and prevalence of foodborne pathogens. PLoS One 13:e0192450. doi: 10.1371/journal.pone.0192450, PMID: 29444134 PMC5812630

[ref79] Lahiri A. Sharif S. Mallick A. I. (2019). Intragastric delivery of recombinant *Lactococcus lactis* displaying ectodomain of influenza matrix protein 2 (M2e) and neuraminidase (NA) induced focused mucosal and systemic immune responses in chickens. Mol. Immunol. 114, 497–512. doi: 10.1016/j.molimm.2019.08.015, PMID: 31518854

[ref80] Lamb-Rosteski J. M. Kalischuk L. D. Inglis G. D. Buret A. G. (2008). Epidermal growth factor inhibits *Campylobacter jejuni*-induced claudin-4 disruption, loss of epithelial barrier function, and Escherichia coli translocation. Infect. Immun. 76, 3390–3398. doi: 10.1128/IAI.01698-07, PMID: 18490463 PMC2493239

[ref81] Lan Y. Verstegen M. Tamminga S. Williams B. (2005). The role of the commensal gut microbial community in broiler chickens. Worlds Poult. Sci. J. 61, 95–104. doi: 10.1079/WPS200445

[ref82] Layton S. L. Morgan M. J. Cole K. Kwon Y. M. Donoghue D. J. Hargis B. M.. (2011). Evaluation of *Salmonella*-vectored *Campylobacter* peptide epitopes for reduction of *Campylobacter jejuni* in broiler chickens. Clin. Vaccine Immunol. 18, 449–454. doi: 10.1128/CVI.00379-10, PMID: 21177910 PMC3067390

[ref83] Lee M. D. Newell D. G. (2006). *Campylobacter* in poultry: filling an ecological niche. Avian Dis. 50, 1–9. doi: 10.1637/7474-111605R.1, PMID: 16617973

[ref84] Ley R. E. Hamady M. Lozupone C. Turnbaugh P. J. Ramey R. R. Bircher J. S.. (2008). Evolution of mammals and their gut microbes. Science 320, 1647–1651. doi: 10.1126/science.1155725, PMID: 18497261 PMC2649005

[ref85] Lin J. (2009). Novel approaches for *Campylobacter* control in poultry. Foodborne Pathog. Dis. 6, 755–765. doi: 10.1089/fpd.2008.0247, PMID: 19425824 PMC3145176

[ref86] Loc Carrillo C. Atterbury R. J. El-Shibiny A. Connerton P. L. Dillon E. Scott A.. (2005). Bacteriophage therapy to reduce *Campylobacter jejuni* colonization of broiler chickens. Appl. Environ. Microbiol. 71, 6554–6563. doi: 10.1128/AEM.71.11.6554-6563.2005, PMID: 16269681 PMC1287621

[ref87] Looft T. Johnson T. A. Allen H. K. Bayles D. O. Alt D. P. Stedtfeld R. D.. (2012). In-feed antibiotic effects on the swine intestinal microbiome. Proc. Natl. Acad. Sci. U. S. A. 109, 1691–1696. doi: 10.1073/pnas.1120238109, PMID: 22307632 PMC3277147

[ref88] Lopetuso L. R. Scaldaferri F. Petito V. Gasbarrini A. (2013). Commensal *Clostridia*: leading players in the maintenance of gut homeostasis. Gut Pathog. 5:23. doi: 10.1186/1757-4749-5-23, PMID: 23941657 PMC3751348

[ref89] Lutful Kabir S. M. (2009). The role of probiotics in the poultry industry. Int. J. Mol. Sci. 10, 3531–3546. doi: 10.3390/ijms10083531, PMID: 20111681 PMC2812824

[ref90] Ma L. Xia Y. Li B. Yang Y. Li L. G. Tiedje J. M.. (2016). Metagenomic Assembly Reveals Hosts of Antibiotic Resistance Genes and the Shared Resistome in Pig, Chicken, and Human Feces. Environ. Sci. Technol. 50, 420–427. doi: 10.1021/acs.est.5b03522, PMID: 26650334

[ref91] Mandal R. K. Jiang T. Wideman R. F. Jr. Lohrmann T. Kwon Y. M. (2020). Microbiota Analysis of Chickens Raised under Stressed Conditions. Front. Vet. Sci. 7:482637. doi: 10.3389/fvets.2020.482637, PMID: 33134343 PMC7575692

[ref92] Manes-Lazaro R. Van Diemen P. M. Pin C. Mayer M. J. Stevens M. P. Narbad A. (2017). Administration of *Lactobacillus johnsonii* FI9785 to chickens affects colonisation by *Campylobacter jejuni* and the intestinal microbiota. Br. Poult. Sci. 58, 373–381. doi: 10.1080/00071668.2017.1307322, PMID: 28318296

[ref93] Maougal R. T. Bargaz A. Sahel C. Amenc L. Djekoun A. Plassard C.. (2014). Localization of the *Bacillus subtilis* beta-propeller phytase transcripts in nodulated roots of *Phaseolus vulgaris* supplied with phytate. Planta 239, 901–908. doi: 10.1007/s00425-013-2023-9, PMID: 24407511

[ref94] Maron D. F. Smith T. J. Nachman K. E. (2013). Restrictions on antimicrobial use in food animal production: an international regulatory and economic survey. Glob. Health 9:48. doi: 10.1186/1744-8603-9-48, PMID: 24131666 PMC3853314

[ref95] Martinez E. A. Babot J. D. Lorenzo-Pisarello M. J. Apella M. C. Chaia A. P. (2016). Feed supplementation with avian *Propionibacterium acidipropionici* contributes to mucosa development in early stages of rearing broiler chickens. Benef Microbes 7, 687–698. doi: 10.3920/BM2016.0077, PMID: 27680209

[ref96] Medina M. Vintini E. Villena J. Raya R. Alvarez S. (2010). *Lactococcus lactis* as an adjuvant and delivery vehicle of antigens against pneumococcal respiratory infections. Bioeng. Bugs 1, 313–325. doi: 10.4161/bbug.1.5.12086, PMID: 21326831 PMC3037581

[ref97] Meijerink N. Kers J. G. Velkers F. C. van Haarlem D. A. Lamot D. M. de Oliveira J. E.. (2020). Early Life Inoculation With Adult-Derived Microbiota Accelerates Maturation of Intestinal Microbiota and Enhances NK Cell Activation in Broiler Chickens. Front. Vet. Sci. 7:584561. doi: 10.3389/fvets.2020.584561, PMID: 33330708 PMC7710667

[ref98] Meng H. Zhang Y. Zhao L. Zhao W. He C. Honaker C. F.. (2014). Body weight selection affects quantitative genetic correlated responses in gut microbiota. PLoS One 9:e89862. doi: 10.1371/journal.pone.0089862, PMID: 24608294 PMC3946484

[ref99] Mordor and Intelligence (2018). Europe poultry feed market - segmented by animal, ingredients, supplements, and country - growth, trends, and forecast (2018–2023). Available at: https://www.mordorintelligence.com/industry-reports

[ref100] Morgun A. Dzutsev A. Dong X. Greer R. L. Sexton D. J. Ravel J.. (2015). Uncovering effects of antibiotics on the host and microbiota using transkingdom gene networks. Gut 64, 1732–1743. doi: 10.1136/gutjnl-2014-308820, PMID: 25614621 PMC5166700

[ref101] Morishita T. Y. Aye P. P. Harr B. S. Cobb C. W. Clifford J. R. (1997). Evaluation of an avian-specific probiotic to reduce the colonization and shedding of *Campylobacter jejuni i*n broilers. Avian Dis. 41, 850–855. doi: 10.2307/1592338, PMID: 9454918

[ref102] Mortada M. Cosby D. E. Akerele G. Ramadan N. Oxford J. Shanmugasundaram R.. (2021). Characterizing the immune response of chickens to *Campylobacter jejuni* (Strain A74C). PLoS One 16:e0247080. doi: 10.1371/journal.pone.0247080, PMID: 33720955 PMC7959354

[ref103] Mortada M. Cosby D. E. Shanmugasundaram R. Selvaraj R. K. (2020). *In vivo* and *in vitro* assessment of commercial probiotic and organic acid feed additives in broilers challenged with *Campylobacter coli*. J. Appl. Poult. Res. 29, 435–446. doi: 10.1016/j.japr.2020.02.001

[ref104] Mueller S. Saunier K. Hanisch C. Norin E. Alm L. Midtvedt T.. (2006). Differences in fecal microbiota in different European study populations in relation to age, gender, and country: a cross-sectional study. Appl. Environ. Microbiol. 72, 1027–1033. doi: 10.1128/AEM.72.2.1027-1033.2006, PMID: 16461645 PMC1392899

[ref105] Muller A. Thomas G. H. Horler R. Brannigan J. A. Blagova E. Levdikov V. M.. (2005). An ATP-binding cassette-type cysteine transporter in *Campylobacter jejuni* inferred from the structure of an extracytoplasmic solute receptor protein. Mol. Microbiol. 57, 143–155. doi: 10.1111/j.1365-2958.2005.04691.x, PMID: 15948956

[ref106] Nakphaichit M. Thanomwongwattana S. Phraephaisarn C. Sakamoto N. Keawsompong S. Nakayama J.. (2011). The effect of including *Lactobacillus reuteri* KUB-AC5 during post-hatch feeding on the growth and ileum microbiota of broiler chickens. Poult. Sci. 90, 2753–2765. doi: 10.3382/ps.2011-01637, PMID: 22080014

[ref107] Neal-McKinney J. M. Lu X. Duong T. Larson C. L. Call D. R. Shah D. H.. (2012). Production of organic acids by probiotic lactobacilli can be used to reduce pathogen load in poultry. PLoS One 7:e43928. doi: 10.1371/journal.pone.0043928, PMID: 22962594 PMC3433458

[ref108] Newell D. G. Elvers K. T. Dopfer D. Hansson I. Jones P. James S.. (2011). Biosecurity-based interventions and strategies to reduce *Campylobacter* spp. on poultry farms. Appl. Environ. Microbiol. 77, 8605–8614. doi: 10.1128/AEM.01090-10, PMID: 21984249 PMC3233073

[ref109] Newsted D. Fallahi F. Golshani A. Azizi A. (2015). Advances and challenges in mucosal adjuvant technology. Vaccine 33, 2399–2405. doi: 10.1016/j.vaccine.2015.03.096, PMID: 25865473

[ref110] Ng S. C. Hart A. L. Kamm M. A. Stagg A. J. Knight S. C. (2009). Mechanisms of action of probiotics: recent advances. Inflamm. Bowel Dis. 15, 300–310. doi: 10.1002/ibd.20602, PMID: 18626975

[ref111] Nishiyama K. Nakazato A. Ueno S. Seto Y. Kakuda T. Takai S.. (2015). Cell surface-associated aggregation-promoting factor from *Lactobacillus gasseri* SBT2055 facilitates host colonization and competitive exclusion of *Campylobacter jejuni*. Mol. Microbiol. 98, 712–726. doi: 10.1111/mmi.13153, PMID: 26239091

[ref112] Nishiyama K. Seto Y. Yoshioka K. Kakuda T. Takai S. Yamamoto Y.. (2014). *Lactobacillus gasseri* SBT2055 reduces infection by and colonization of *Campylobacter jejuni*. PLoS One 9:e108827. doi: 10.1371/journal.pone.0108827, PMID: 25264604 PMC4181664

[ref113] Nobel Y. R. Cox L. M. Kirigin F. F. Bokulich N. A. Yamanishi S. Teitler I.. (2015). Metabolic and metagenomic outcomes from early-life pulsed antibiotic treatment. Nat. Commun. 6:7486. doi: 10.1038/ncomms8486, PMID: 26123276 PMC4491183

[ref114] Nothaft H. Davis B. Lock Y. Y. Perez-Munoz M. E. Vinogradov E. Walter J.. (2016). Engineering the *Campylobacter jejuni* N-glycan to create an effective chicken vaccine. Sci. Rep. 6:26511. doi: 10.1038/srep26511, PMID: 27221144 PMC4879521

[ref115] Oakley B. B. Buhr R. J. Ritz C. W. Kiepper B. H. Berrang M. E. Seal B. S.. (2014a). Successional changes in the chicken cecal microbiome during 42 days of growth are independent of organic acid feed additives. BMC Vet. Res. 10:282. doi: 10.1186/s12917-014-0282-8, PMID: 25427406 PMC4251860

[ref116] Oakley B. B. Lillehoj H. S. Kogut M. H. Kim W. K. Maurer J. J. Pedroso A.. (2014b). The chicken gastrointestinal microbiome. FEMS Microbiol. Lett. 360, 100–112. doi: 10.1111/1574-6968.12608 25263745

[ref117] Oh J. K. Pajarillo E. A. B. Chae J. P. Kim I. H. Yang D. S. Kang D. K. (2017). Effects of Bacillus subtilis CSL2 on the composition and functional diversity of the faecal microbiota of broiler chickens challenged with *Salmonella Gallinarum*. J. Anim. Sci. Biotechnol. 8:1. doi: 10.1186/s40104-016-0130-8, PMID: 28070331 PMC5215103

[ref118] Pan D. Yu Z. (2014). Intestinal microbiome of poultry and its interaction with host and diet. Gut Microbes 5, 108–119. doi: 10.4161/gmic.26945, PMID: 24256702 PMC4049927

[ref119] Pandit R. J. Hinsu A. T. Patel N. V. Koringa P. G. Jakhesara S. J. Thakkar J. R.. (2018). Microbial diversity and community composition of caecal microbiota in commercial and indigenous Indian chickens determined using 16s rDNA amplicon sequencing. Microbiome 6:115. doi: 10.1186/s40168-018-0501-9, PMID: 29935540 PMC6015460

[ref120] Patuzzi I. Orsini M. Cibin V. Petrin S. Mastrorilli E. Tiengo A.. (2021). The Interplay between *Campylobacter* and the Caecal Microbial Community of Commercial Broiler Chickens over Time. Microorganisms 9:221. doi: 10.3390/microorganisms9020221, PMID: 33499060 PMC7911313

[ref121] Pedroso A. A. Menten J. F. M. Lambais M. R. (2005). The structure of bacterial community in the intestines of newly hatched chicks. J. Appl. Poult. Res. 14, 232–237. doi: 10.1093/japr/14.2.232

[ref122] Peng Q. Zeng X. F. Zhu J. L. Wang S. Liu X. T. Hou C. L.. (2016). Effects of dietary *Lactobacillus plantarum* B1 on growth performance, intestinal microbiota, and short chain fatty acid profiles in broiler chickens. Poult. Sci. 95, 893–900. doi: 10.3382/ps/pev435, PMID: 26772658

[ref123] Reich F. Atanassova V. Haunhorst E. Klein G. (2008). The effects of *Campylobacter* numbers in caeca on the contamination of broiler carcasses with *Campylobacter*. Int. J. Food Microbiol. 127, 116–120. doi: 10.1016/j.ijfoodmicro.2008.06.018, PMID: 18657873

[ref124] Renouf M. Hendrich S. (2011). *Bacteroides uniformis* is a putative bacterial species associated with the degradation of the isoflavone genistein in human feces. J. Nutr. 141, 1120–1126. doi: 10.3945/jn.111.140988, PMID: 21525249

[ref125] Richards P. J. Flaujac Lafontaine G. M. Connerton P. L. Liang L. Asiani K. Fish N. M.. (2020). Galacto-Oligosaccharides Modulate the Juvenile Gut Microbiome and Innate Immunity To Improve Broiler Chicken Performance. mSystems 5:e00827-19. doi: 10.1128/mSystems.00827-19, PMID: 31937680 PMC6967391

[ref126] Richards P. Fothergill J. Bernardeau M. Wigley P. (2019). Development of the Caecal Microbiota in Three Broiler Breeds. Front. Vet. Sci. 6:201. doi: 10.3389/fvets.2019.00201, PMID: 31294039 PMC6603203

[ref127] Rinttila T. Apajalahti J. (2013). Intestinal microbiota and metabolites-Implications for broiler chicken health and performance. J. Appl. Poult. Res. 22, 647–658. doi: 10.3382/japr.2013-00742

[ref128] Robyn J. Rasschaert G. Pasmans F. Heyndrickx M. (2015). Thermotolerant *Campylobacter* during Broiler Rearing: Risk Factors and Intervention. Compr. Rev. Food Sci. Food Saf. 14, 81–105. doi: 10.1111/1541-4337.12124, PMID: 33401809

[ref129] Rocha T. S. Baptista A. A. Donato T. C. Milbradt E. L. Okamoto A. S. Rodrigues J. C.. (2012). Evaluation of *in vitro* and *in vivo* adhesion and immunomodulatory effect of *Lactobacillus* species strains isolated from chickens. Poult. Sci. 91, 362–369. doi: 10.3382/ps.2011-01803, PMID: 22252349

[ref130] Rosenquist H. Nielsen N. L. Sommer H. M. Norrung B. Christensen B. B. (2003). Quantitative risk assessment of human campylobacteriosis associated with thermophilic *Campylobacter* species in chickens. Int. J. Food Microbiol. 83, 87–103. doi: 10.1016/S0168-1605(02)00317-3, PMID: 12672595

[ref131] Saez D. Fernandez P. Rivera A. Andrews E. Onate A. (2012). Oral immunization of mice with recombinant *Lactococcus lactis* expressing Cu,Zn superoxide dismutase of *Brucella abortus* triggers protective immunity. Vaccine 30, 1283–1290. doi: 10.1016/j.vaccine.2011.12.088, PMID: 22222868

[ref132] Sahin O. Kassem I. I. Shen Z. Lin J. Rajashekara G. Zhang Q. (2015). *Campylobacter* in Poultry: Ecology and Potential Interventions. Avian Dis. 59, 185–200. doi: 10.1637/11072-032315-Review, PMID: 26473668

[ref133] Sahin O. Luo N. Huang S. Zhang Q. (2003). Effect of *Campylobacter*-specific maternal antibodies on *Campylobacter jejuni* colonization in young chickens. Appl. Environ. Microbiol. 69, 5372–5379. doi: 10.1128/AEM.69.9.5372-5379.2003, PMID: 12957925 PMC194908

[ref134] Saint-Cyr M. J. Guyard-Nicodeme M. Messaoudi S. Chemaly M. Cappelier J. M. Dousset X.. (2016). Recent Advances in screening of Anti-*Campylobacter* Activity in Probiotics for Use in Poultry. Front. Microbiol. 7:553. doi: 10.3389/fmicb.2016.00553, PMID: 27303366 PMC4885830

[ref135] Salaheen S. Kim S. W. Haley B. J. Van Kessel J. A. S. Biswas D. (2017). Alternative Growth Promoters Modulate Broiler Gut Microbiome and Enhance Body Weight Gain. Front. Microbiol. 8:2088. doi: 10.3389/fmicb.2017.02088, PMID: 29123512 PMC5662582

[ref136] Salim H. M. Kang H. K. Akter N. Kim D. W. Kim J. H. Kim M. J.. (2013). Supplementation of direct-fed microbials as an alternative to antibiotic on growth performance, immune response, cecal microbial population, and ileal morphology of broiler chickens. Poult. Sci. 92, 2084–2090. doi: 10.3382/ps.2012-02947, PMID: 23873556

[ref137] Salyers A. A. Gupta A. Wang Y. (2004). Human intestinal bacteria as reservoirs for antibiotic resistance genes. Trends Microbiol. 12, 412–416. doi: 10.1016/j.tim.2004.07.004, PMID: 15337162

[ref138] Sarao L. K. Arora M. (2017). Probiotics, prebiotics, and microencapsulation: a review. Crit. Rev. Food Sci. Nutr. 57, 344–371. doi: 10.1080/10408398.2014.887055, PMID: 25848935

[ref139] Scanes C. G. (2007). The global importance of poultry. Poult. Sci. 86, 1057–1058. doi: 10.1093/ps/86.6.1057, PMID: 17495072

[ref140] Sefcova M. Larrea-Alvarez M. Larrea-Alvarez C. Karaffova V. Revajova V. Gancarcikova S.. (2020a). *Lactobacillus fermentum* Administration Modulates Cytokine Expression and Lymphocyte Subpopulation Levels in Broiler Chickens Challenged with *Campylobacter coli*. Foodborne Pathog. Dis. 17, 485–493. doi: 10.1089/fpd.2019.2739, PMID: 31977245

[ref141] Sefcova M. Larrea-Alvarez M. Larrea-Alvarez C. Revajova V. Karaffova V. Koscova J.. (2020b). Effects of *Lactobacillus Fermentum* Supplementation on Body Weight and Pro-Inflammatory Cytokine Expression in *Campylobacter Jejuni*-Challenged Chickens. Vet. Sci. 7:121. doi: 10.3390/vetsci7030121, PMID: 32872452 PMC7557755

[ref142] Sekirov I. Russell S. L. Antunes L. C. Finlay B. B. (2010). Gut microbiota in health and disease. Physiol. Rev. 90, 859–904. doi: 10.1152/physrev.00045.2009, PMID: 20664075

[ref143] Sergeant M. J. Constantinidou C. Cogan T. A. Bedford M. R. Penn C. W. Pallen M. J. (2014). Extensive microbial and functional diversity within the chicken cecal microbiome. PLoS One 9:e91941. doi: 10.1371/journal.pone.0091941, PMID: 24657972 PMC3962364

[ref144] Sikic Pogacar M. Langerholc T. Micetic-Turk D. Mozina S. S. Klancnik A. (2020). Effect of *Lactobacillus* spp. on adhesion, invasion, and translocation of *Campylobacter jejuni* in chicken and pig small-intestinal epithelial cell lines. BMC Vet. Res. 16:34. doi: 10.1186/s12917-020-2238-5, PMID: 32013961 PMC6998324

[ref145] Silva J. Leite D. Fernandes M. Mena C. Gibbs P. A. Teixeira P. (2011). *Campylobacter* spp. as a Foodborne Pathogen: A Review. Front. Microbiol. 2:200. doi: 10.3389/fmicb.2011.00200, PMID: 21991264 PMC3180643

[ref146] Singh K. M. Shah T. Deshpande S. Jakhesara S. J. Koringa P. G. Rank D. N.. (2012). High through put 16S rRNA gene-based pyrosequencing analysis of the fecal microbiota of high FCR and low FCR broiler growers. Mol. Biol. Rep. 39, 10595–10602. doi: 10.1007/s11033-012-1947-7, PMID: 23053958

[ref147] Singh K. M. Shah T. M. Reddy B. Deshpande S. Rank D. N. Joshi C. G. (2014). Taxonomic and gene-centric metagenomics of the fecal microbiome of low and high feed conversion ratio (FCR) broilers. J. Appl. Genet. 55, 145–154. doi: 10.1007/s13353-013-0179-4, PMID: 24136777

[ref148] Smialek M. Burchardt S. Koncicki A. (2018). The influence of probiotic supplementation in broiler chickens on population and carcass contamination with *Campylobacter* spp. - field study. Res. Vet. Sci. 118, 312–316. doi: 10.1016/j.rvsc.2018.03.009, PMID: 29567598

[ref149] Sommer F. Backhed F. (2013). The gut microbiota--masters of host development and physiology. Nat. Rev. Microbiol. 11, 227–238. doi: 10.1038/nrmicro2974, PMID: 23435359

[ref150] Spivey M. A. Dunn-Horrocks S. L. Duong T. (2014). Epithelial cell adhesion and gastrointestinal colonization of *Lactobacillus* in poultry. Poult. Sci. 93, 2910–2919. doi: 10.3382/ps.2014-04076, PMID: 25239531

[ref151] Stanley D. Geier M. S. Denman S. E. Haring V. R. Crowley T. M. Hughes R. J.. (2013). Identification of chicken intestinal microbiota correlated with the efficiency of energy extraction from feed. Vet. Microbiol. 164, 85–92. doi: 10.1016/j.vetmic.2013.01.030, PMID: 23434185

[ref152] Stanley D. Hughes R. J. Geier M. S. Moore R. J. (2016). Bacteria within the Gastrointestinal Tract Microbiota Correlated with Improved Growth and Feed Conversion: Challenges Presented for the Identification of Performance Enhancing Probiotic Bacteria. Front. Microbiol. 7:187. doi: 10.3389/fmicb.2016.00187, PMID: 26925052 PMC4760072

[ref153] Stern N. J. Svetoch E. A. Eruslanov B. V. Kovalev Y. N. Volodina L. I. Perelygin V. V.. (2005). *Paenibacillus polymyxa* purified bacteriocin to control *Campylobacter jejuni* in chickens. J. Food Prot. 68, 1450–1453. doi: 10.4315/0362-028X-68.7.1450, PMID: 16013385

[ref154] Stern N. J. Svetoch E. A. Eruslanov B. V. Perelygin V. V. Mitsevich E. V. Mitsevich I. P.. (2006). Isolation of a *Lactobacillus salivarius* strain and purification of its bacteriocin, which is inhibitory to *Campylobacter jejuni* in the chicken gastrointestinal system. Antimicrob. Agents Chemother. 50, 3111–3116. doi: 10.1128/AAC.00259-06, PMID: 16940109 PMC1563535

[ref155] Sun N. Zhang R. Duan G. Peng X. Wang C. Fan Q.. (2017). An engineered food-grade *Lactococcus lactis* strain for production and delivery of heat-labile enterotoxin B subunit to mucosal sites. BMC Biotechnol. 17:25. doi: 10.1186/s12896-017-0345-6, PMID: 28264682 PMC5339977

[ref156] Sureshkumar S. Lee H. C. Jung S. K. Kim D. Oh K. B. Yang H.. (2021). Inclusion of *Lactobacillus salivarius* strain revealed a positive effect on improving growth performance, fecal microbiota and immunological responses in chicken. Arch. Microbiol. 203, 847–853. doi: 10.1007/s00203-020-02088-3, PMID: 33068123

[ref157] Swaggerty C. L. Pevzner I. Y. He H. Genovese K. J. Kogut M. H. (2017). Selection for pro-inflammatory mediators produces chickens more resistant to *Campylobacter jejuni*. Poult. Sci. 96, 1623–1627. doi: 10.3382/ps/pew465, PMID: 28339707

[ref158] Taha-Abdelaziz K. Astill J. Kulkarni R. R. Read L. R. Najarian A. Farber J. M.. (2019). In vitro assessment of immunomodulatory and anti-*Campylobacter* activities of probiotic lactobacilli. Sci. Rep. 9:17903. doi: 10.1038/s41598-019-54494-3, PMID: 31784645 PMC6884649

[ref159] Tarradas J. Tous N. Esteve-Garcia E. Brufau A. J. (2020). The Control of Intestinal Inflammation: A Major Objective in the Research of Probiotic Strains as Alternatives to Antibiotic Growth Promoters in Poultry. Microorganisms 8:148. doi: 10.3390/microorganisms8020148, PMID: 31973199 PMC7074883

[ref160] Thibodeau A. Fravalo P. Yergeau E. Arsenault J. Lahaye L. Letellier A. (2015). Chicken caecal Microbiome Modifications Induced by *Campylobacter jejuni* Colonization and by a Non-Antibiotic Feed Additive. PLoS One 10:e0131978. doi: 10.1371/journal.pone.0131978, PMID: 26161743 PMC4498643

[ref161] Timmerman H. M. Veldman A. van den Elsen E. Rombouts F. M. Beynen A. C. (2006). Mortality and growth performance of broilers given drinking water supplemented with chicken-specific probiotics. Poult. Sci. 85, 1383–1388. doi: 10.1093/ps/85.8.1383, PMID: 16903468

[ref162] Tsubokura K. Berndtson E. Bogstedt A. Kaijser B. Kim M. Ozeki M.. (1997). Oral administration of antibodies as prophylaxis and therapy in *Campylobacter jejuni*-infected chickens. Clin. Exp. Immunol. 108, 451–455. doi: 10.1046/j.1365-2249.1997.3901288.x, PMID: 9182891 PMC1904686

[ref163] Turnbaugh P. J. Ley R. E. Mahowald M. A. Magrini V. Mardis E. R. Gordon J. I. (2006). An obesity-associated gut microbiome with increased capacity for energy harvest. Nature 444, 1027–1031. doi: 10.1038/nature05414, PMID: 17183312

[ref164] van der Wielen P. W. Keuzenkamp D. A. Lipman L. J. van Knapen F. Biesterveld S. (2002). Spatial and temporal variation of the intestinal bacterial community in commercially raised broiler chickens during growth. Microb. Ecol. 44, 286–293. doi: 10.1007/s00248-002-2015-y, PMID: 12219265

[ref165] Van Deun K. Pasmans F. Van Immerseel F. Ducatelle R. Haesebrouck F. (2008). Butyrate protects Caco-2 cells from *Campylobacter jejuni* invasion and translocation. Br. J. Nutr. 100, 480–484. doi: 10.1017/S0007114508921693, PMID: 18275629

[ref166] Vandeputte J. Martel A. Canessa S. Van Rysselberghe N. De Zutter L. Heyndrickx M.. (2019). Reducing *Campylobacter jejuni* colonization in broiler chickens by in-feed supplementation with hyperimmune egg yolk antibodies. Sci. Rep. 9:8931. doi: 10.1038/s41598-019-45380-z, PMID: 31222043 PMC6586802

[ref167] Wagenaar J. A. Van Bergen M. A. Mueller M. A. Wassenaar T. M. Carlton R. M. (2005). Phage therapy reduces *Campylobacter jejuni* colonization in broilers. Vet. Microbiol. 109, 275–283. doi: 10.1016/j.vetmic.2005.06.002, PMID: 16024187

[ref168] Wang Y. Wang J. Dai W. (2011). Use of GFP to trace the colonization of *Lactococcus lactis* WH-C1 in the gastrointestinal tract of mice. J. Microbiol. Methods 86, 390–392. doi: 10.1016/j.mimet.2011.06.009, PMID: 21704659

[ref169] Wang C. Zhou H. Guo F. Yang B. Su X. Lin J.. (2020). Oral Immunization of Chickens with *Lactococcus lactis* Expressing cjaA Temporarily reduces *Campylobacter jejuni* Colonization. Foodborne Pathog. Dis. 17, 366–372. doi: 10.1089/fpd.2019.2727, PMID: 31718285

[ref170] Wells J. M. Wilson P. W. Norton P. M. Gasson M. J. Le Page R. W. (1993). *Lactococcus lactis*: high-level expression of tetanus toxin fragment C and protection against lethal challenge. Mol. Microbiol. 8, 1155–1162. doi: 10.1111/j.1365-2958.1993.tb01660.x, PMID: 8361360

[ref171] Williams L. K. Sait L. C. Trantham E. K. Cogan T. A. Humphrey T. J. (2013). *Campylobacter* infection has different outcomes in fast- and slow-growing broiler chickens. Avian Dis. 57, 238–241. doi: 10.1637/10442-110212-Reg.1, PMID: 24689180

[ref172] Witzig M. Carminha-Silva A. Green-Engert R. Hoelzle K. Zeller E. Seifert J.. (2015). Spatial Variation of the Gut Microbiota in Broiler Chickens as Affected by Dietary Available Phosphorus and Assessed by T-RFLP Analysis and 454 Pyrosequencing. PLoS One 10:e0143442. doi: 10.1371/journal.pone.0143442, PMID: 26588075 PMC4654470

[ref173] Wrzosek L. Miquel S. Noordine M. L. Bouet S. Joncquel C.-C. M. Robert V.. (2013). *Bacteroides thetaiotaomicron* and *Faecalibacterium prausnitzii* influence the production of mucus glycans and the development of goblet cells in the colonic epithelium of a gnotobiotic model rodent. BMC Biol. 11:61. doi: 10.1186/1741-7007-11-61, PMID: 23692866 PMC3673873

[ref174] Wyszynska A. Zycka J. Godlewska R. Jagusztyn-Krynicka E. K. (2008). The *Campylobacter jejuni/coli* cjaA (cj0982c) gene encodes an N-glycosylated lipoprotein localized in the inner membrane. Curr. Microbiol. 57, 181–188. doi: 10.1007/s00284-008-9171-3, PMID: 18584243

[ref175] Yegani M. Korver D. R. (2008). Factors affecting intestinal health in poultry. Poult. Sci. 87, 2052–2063. doi: 10.3382/ps.2008-00091, PMID: 18809868 PMC7107194

[ref176] Zommiti M. Almohammed H. Ferchichi M. (2016). Purification and Characterization of a Novel Anti-*Campylobacter* Bacteriocin Produced by *Lactobacillus curvatus* DN317. Probiotics Antimicrob. Proteins 8, 191–201. doi: 10.1007/s12602-016-9237-7, PMID: 27812926

